# Chromium-ruthenium oxide solid solution electrocatalyst for highly efficient oxygen evolution reaction in acidic media

**DOI:** 10.1038/s41467-018-08144-3

**Published:** 2019-01-11

**Authors:** Yichao Lin, Ziqi Tian, Linjuan Zhang, Jingyuan Ma, Zheng Jiang, Benjamin J. Deibert, Ruixiang Ge, Liang Chen

**Affiliations:** 1 Ningbo Institute of Materials Technology and Engineering, Chinese Academy of Sciences, 315201 Ningbo, Zhejiang China; 2 University of Chinese Academy of Sciences, 100049 Beijing, China; 3 Shanghai Institute of Applied Physics, Chinese Academy of Sciences, 201800 Shanghai, China; 4 Shanghai Synchrotron Radiation Facility, Shanghai Institute of Applied Physics, Chinese Academy of Sciences, 201204 Shanghai, China; 5 Department of Chemistry and Chemical Biology, Rutgers, The State University of New Jersey, Piscataway, NJ 08854 USA

## Abstract

The development of active, acid-stable and low-cost electrocatalysts for oxygen evolution reaction is urgent and challenging. Herein we report an Iridium-free and low ruthenium-content oxide material (Cr_0.6_Ru_0.4_O_2_) derived from metal-organic framework with remarkable oxygen evolution reaction performance in acidic condition. It shows a record low overpotential of 178 mV at 10 mA cm^−2^ and maintains the excellent performance throughout the 10 h chronopotentiometry test at a constant current of 10 mA cm^−2^ in 0.5 M H_2_SO_4_ solution. Density functional theory calculations further revealed the intrinsic mechanism for the exceptional oxygen evolution reaction performance, highlighting the influence of chromium promoter on the enhancement in both activity and stability.

## Abstract (web summary)

H_2_ generated from water electrolysis is a promising, carbon-neutral fuel, but sluggish oxidation kinetics and poor catalyst stability in acid limits the chemistry. Here, authors prepare solid-solution catalysts based on ruthenium-chromium oxides that are stable and active for acidic water oxidation.

## Introduction

Oxygen evolution reaction (OER) or the water oxidation plays a key role in clean energy technologies, including hydrogen production through water electrolysis, electrochemical or photoelectrochemical CO_2_ reduction and reversible fuel cells for production of clean electricity^1–3^. Essentially, the process of OER is a four electron and four proton coupled electrochemical reaction, demanding a higher energy (i.e., higher overpotential) to overcome the kinetic barrier than the hydrogen evolution reaction (HER), which is a two electron-transfer reaction. In the past decades, substantial research effort has been devoted to the design and development of OER electrocatalysts with enhanced electrode kinetics and stability. To date, various OER catalysts, such as transition metal oxides^4–6^, perovskite^7,8^, and layered structure materials^9,10^, have been reported. However, these OER electrocatalysts still suffer from sluggish kinetics and/or low stability in acidic media. Compared with alkaline conditions, OER catalysis under acidic conditions is much more preferable because acidic electrolyte has higher ionic conductivity and fewer unfavorable reactions^11,12^. In addition, commercially available water electrolysis assemblies use cation exchange membrane, e.g., Nafion, as the ionic conductor, which requires OER to be operated in acidic environment. Unfortunately, most of the known active metal oxides cannot survive under harsh acidic operating conditions. Currently, rutile-structured ruthenium (Ru) and iridium (Ir) oxides are the two best catalysts for OER in acidic media^13–15^. It is widely accepted that RuO_2_ has higher activity but lower stability than IrO_2_^16–18^. Thus, to develop OER catalysts with both high activity and stability, the use of mixed phase or solid solution of RuO_2_ and IrO_2_ has been investigated^19–22^. Very recently, three new types of Ir-based double perovskites^23^, multiphase IrNiO_x_ or IrO_x_/SrIrO_3_^24,25^, and pyrochlores-structured Ir-based oxides^26^, were reported to be active and relatively stable toward OER in acidic media. However, we note that little attention has been paid to design cheaper Ru-based electrocatalysts, particularly with low Ru-content, for OER in acidic condition^27^. Indeed, it is desirable to modulate the electronic structure by replacing part of Ru with suitable transition metals in order to improve the OER activity. Furthermore, the replacement by cheaper transition metal is also advantageous in terms of cost.

Metal-organic frameworks (MOFs), a unique type of porous materials with ultrahigh porosity, tunable pore sizes and morphology, and well-characterized crystalline architectures, have emerged as excellent templates or platforms for preparing electrocatalysts with high performance, such as N-doped porous carbon, metal oxide nanocomposites^3,28^. In light of these successful studies, we propose to design Ru-based electrocatalysts based on MOF templates, which can make use of the porosity to load Ru sources and the original metal node as promoter. Herein, we present a low-cost Ir-free rutile-structured chromium-ruthenium oxide electrocatalyst (i.e., Cr_0.6_Ru_0.4_O_2_) derived from MIL-101 (Cr) which exhibits record low overpotential and high stability toward OER in acidic media. We chose MIL-101 (Cr) because of its ultra-high surface area (above 3000 m^2^ g^−1^) and large pore sizes (2.9–3.2 nm) that can facilitate the loading of Ru precursors^29^. Moreover, density functional theory (DFT) calculations suggested that Cr plays a critical role in improving the stability and OER activity by tuning the electronic structure of RuO_2_ phase. The resulting Cr_0.6_Ru_0.4_O_2_ electrocatalyst exhibits an overpotential of 178 mV at 10 mA cm^−2^, a small Tafel slope (58 mV dec^−1^), and stable chronopotentiometric performance under 10 mA cm^−2^ in 0.5 M H_2_SO_4_ solution for 10 h, which outperforms the most active OER electrocatalysts reported to date, such as BaYIrO_6_^23^, IrO_x_/SrIrO_3_^25^, and Y_2_Ru_2_O_7-δ_^27^.

## Results

### Preparation and characterization of RuCl_3_-MIL-101(Cr) and Cr_0.6_Ru_0.4_O_2_

**Fig. 1:**
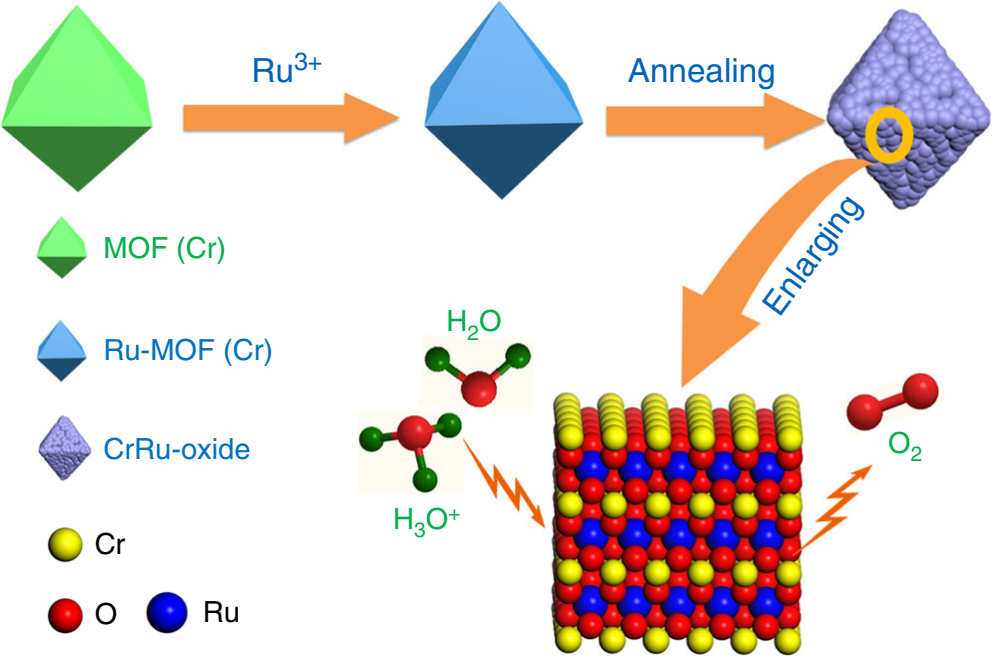
Schematic illustration of the preparation of Cr_0.6_Ru_0.4_O_2_ electrocatalysts for OER application in acid media

**Fig. 2:**
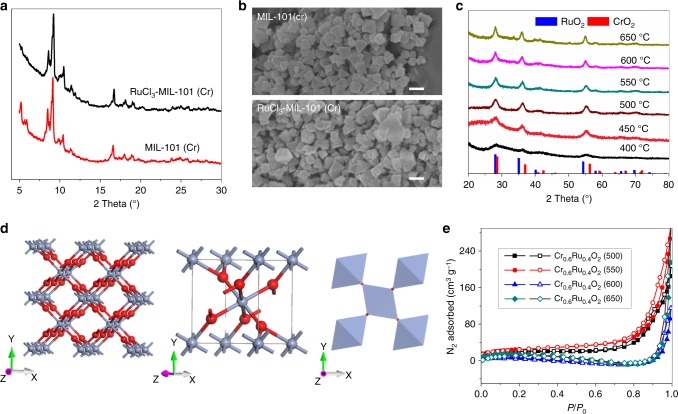
Structural characterizations of RuCl_3_-MIL-101(Cr) and Cr_0.6_Ru_0.4_O_2_ (550). **a**, **b** PXRD patterns and SEM images of MIL-101 (Cr) before and after loading RuCl_3_ (scale bars, 200 nm); **c** PXRD patterns of Cr_0.6_Ru_0.4_O_2_ powders annealed at different temperatures. The reference patterns of CrO_2_ and RuO_2_ were obtained from Jade 2004 (JCPDS No.09-0332 and 43-1027); **d** Crystal structure of Cr_0.6_Ru_0.4_O_2_ (550): (left) packing image, (middle) unit cell, (right) corner shared octahedral MO_6_ structure. Color code: blue (60% Ru, 40% Cr), red (O). **e** 77 K N_2_ adsorption/desorption isotherms of Cr_0.6_Ru_0.4_O_2_ powders annealed at different temperatures

The route to the preparation of RuCl_3_-MIL-101(Cr) precursor and Cr_0.6_Ru_0.4_O_2_ powders is illustrated in Fig. 1. RuCl_3_ was firstly loaded into the pores of MIL-101 (Cr) by means of impregnation. After loading RuCl_3_, the color of MIL-101 (Cr) changed from light green to brown (the color of RuCl_3_) (Supplementary Figure 1), visually indicating the successful loading of RuCl_3_. The resulting RuCl_3_-MIL-101 (Cr) composite was further annealed under air at temperatures between 450 and 600 °C for 4 h to fabricate Cr_0.6_Ru_0.4_O_2_ powders. RuCl_3_-MIL-101 (Cr) was characterized using a combination of power X-ray diffractions (PXRD), scanning electron microscopy (SEM), inductively coupled plasma-mass spectroscopy (ICP-MS) and N_2_ adsorption/desorption measurements at 77 K. As shown in Fig. 2a. PXRD pattern of the resulting RuCl_3_-MIL-101 (Cr) was essentially identical to that of original MIL-101 (Cr), suggesting that the crystalline structure of MIL-101 (Cr) was preserved after loading RuCl_3_. The reduced intensity of the peaks below 7° after loading RuCl_3_ can be attributed to the pore filling of MIL-101 (Cr), which has also been observed in PEI incorporated MIL-101 (Cr)^30^. There was no peak for RuCl_3_, indicating that RuCl_3_ did not crystalize in the pores of MIL-101 (Cr) but was adsorbed on the pore surface. SEM characterization was conducted to analyze the morphology of MIL-101 (Cr) before and after loading RuCl_3_. As displayed in Fig. 2b, MIL-101 (Cr) has an octahedral morphology with small particle size (~100 nm), which can effectively facilitate the diffusion of RuCl_3_ into MIL-101 (Cr) pores. After loading RuCl_3_, morphology change of MIL-101 (Cr) was not observed. ICP-MS was employed to evaluate the loading amount of RuCl_3_ in MIL-101 (Cr). The measured atomic ratio of Cr/Ru was 6:4, corresponding to 37.8 wt% RuCl_3_ content in RuCl_3_-MIL-101 (Cr). N_2_ adsorption/desorption measurements of MIL-101 (Cr) and RuCl_3_-MIL-101 (Cr) were further conducted to evaluate their surface area and pore volume (Supplementary Figure 2). MIL-101 (Cr) exhibited a saturated N_2_ uptake of 1050 cm^3^ g^−1^, which was consistent with values reported in literatures^31,32^. The corresponding pore volume and BET surface area were calculated to be 1.63 cm^3^ g^−1^ and 3373 m^2^ g^−1^, respectively. Upon the loading of RuCl_3_, the pore volume and Brunauer−Emmett−Teller (BET) surface area were decreased to 0.97 cm^3^ g^−1^ and 1783 m^2^ g^−1^, respectively.

**Fig. 3:**
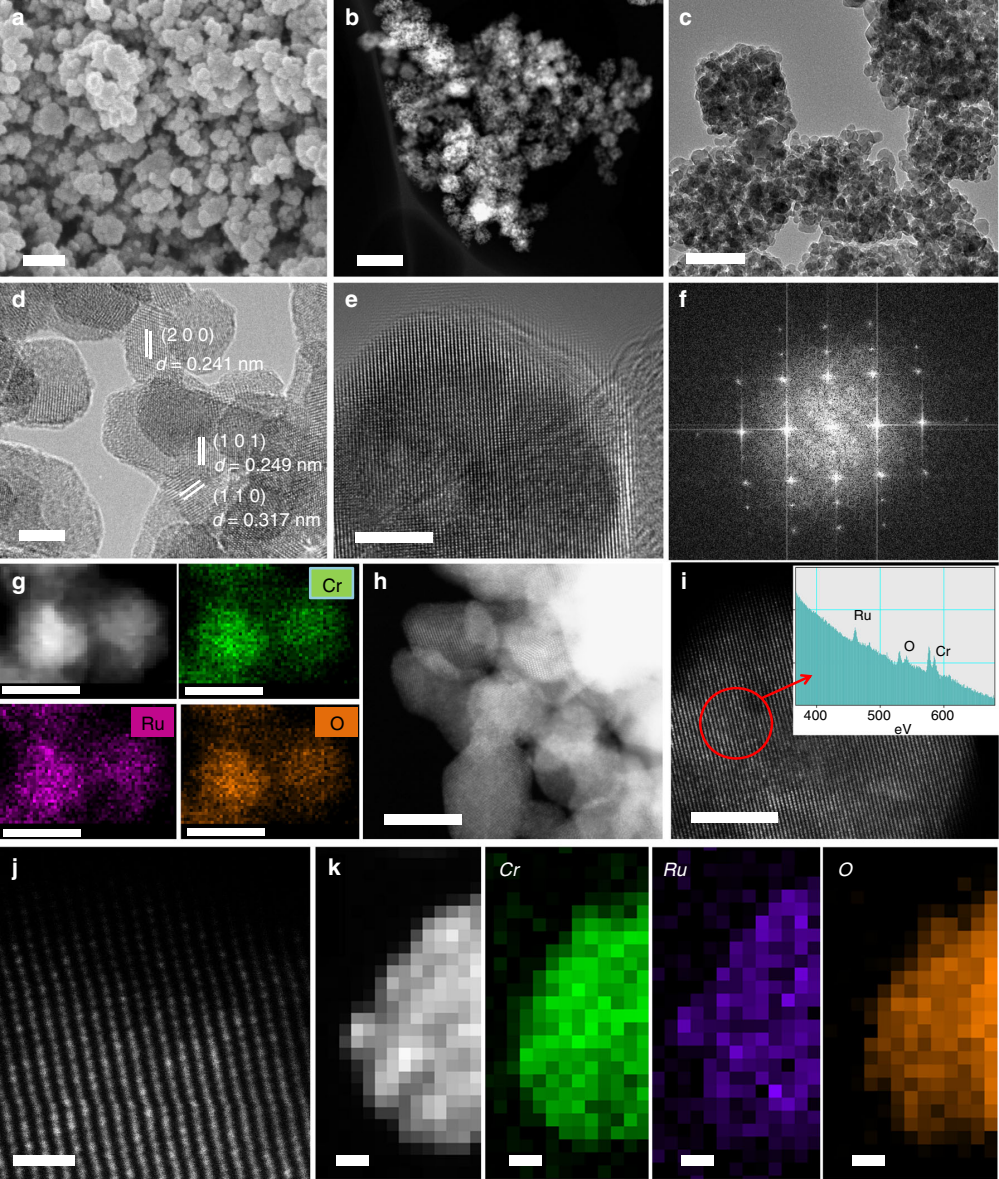
Morphology and elemental mapping images of Cr_0.6_Ru_0.4_O_2_ (550). **a** SEM image (scale bar, 200 nm); **b** Dark field TEM image (scale bar, 200 nm); **c** TEM image (scale bar, 50 nm); **d** HR-TEM image (scale bar, 5 nm); **e** HR-TEM image of a single nanocrystal (scale bar, 5 nm); **f** The corresponding FFT image; **g** HAADF-STEM image, corresponding EDS element mapping showing the distribution of Cr, Ru, and O (scale bars, 10 nm); **h**–**j** atomic-resolution HAADF-STEM images and EELS analysis (inset of i), scale bars: 10, 5, and 1 nm, respectively; **k** EELS maps (scale bars, 1 nm)

Fine powders with the composition of Cr_0.6_Ru_0.4_O_2_ were obtained by annealing RuCl_3_-MIL-101 (Cr) under air for 4 h at a series of temperatures between 400 and 650 °C, denoted as Cr_0.6_Ru_0.4_O_2_ (T, T is the annealing temperature). As shown in the PXRD patterns (Fig. 2c), the increased intensity of peaks with annealing temperature indicates that the higher annealing temperature can lead to better cystallinity of Cr_0.6_Ru_0.4_O_2._ When the annealing temperature was lower than 450 °C, very poor crystalline samples were formed. The PXRD patterns of Cr_0.6_Ru_0.4_O_2_ powders annealed above 500 °C are essentially identical, and can be indexed as a solid solution of rutile CrO_2_ and RuO_2_ with tetragonal system and P42/mnm space group (the refined lattice parameters are listed in Supplementary Table 1 and the standard PXRD patterns of CrO_2_ and RuO_2_ were shown in Supplementary Figure 3a for comparison). The structure of Cr_0.6_Ru_0.4_O_2_ is refined by Rietveld refinement (Supplementary Figure 3b). As shown in Fig. 2d, Cr and Ru atoms are randomly distributed in the metal sites of the Cr_0.6_Ru_0.4_O_2_ lattice. These metal atoms are edge−sharing and octahedrally coordinated to form chains along the [0 0 1] direction. Each chain is connected to four neighboring chains by shared corners. The MO_6_ octahedra are tetragonally distorted, thus these M−O bond distances are not equal. SEM images show that the morphologies of Cr_0.6_Ru_0.4_O_2_ powders became smaller, and their surface became much rougher after annealing (Fig. 3a and Supplementary Figure 4). Transmission electron microscopy (TEM) images indicate that the individual particles are composed of much smaller nanocrystals (~15 nm) (Fig. 3b–d, Supplementary Figures 5-8). High resolution TEM (HR-TEM) image (Fig. 3e) and the corresponding fast Fourier transform (FFT, Fig. 3f) indicate that these nanocrystals are single-crystalline. Between these nanocrystals in a single Cr_0.6_Ru_0.4_O_2_ particle, many mesopores exist, facilitating the mass transfer in the OER process. The N_2_ adsorption/desorption isotherms further confirm that Cr_0.6_Ru_0.4_O_2_ powders are porous with BET surface areas between 50 and 90 m^2^ g^−1^ (Fig. 2e and Supplementary Table 2). Barrett–Joyner–Halenda (BJH) pore size analysis reveals that the pore sizes of Cr_0.6_Ru_0.4_O_2_ particle are larger than 10 nm (generated from the aggregation of nanocrystals in an individual particle as shown in TEM images) and increase with the annealing temperature (Supplementary Figure 9). This trend can be ascribed to the larger volume contraction of Cr_0.6_Ru_0.4_O_2_ nanocrystals within a single particle at higher annealing temperature. High-angle annular dark-field scanning transmission electron microscopy (HAADF-STEM) was employed to analyze the element distribution in a single nanocrystal. The resulting EDS mapping images (Fig. 3g) show that Cr, Ru, and O are uniformly distributed over the entire Cr_0.6_Ru_0.4_O_2_ nanocrystal, demonstrating the formation of a single phase of Cr and Ru oxide solid solution (the mapping images for a wider region are shown in Supplementary Figure 10). In addition, the EDS analysis indicates that Cr/Ru ratio is 0.56:0.44, generally consistent with the ICP-MS result (Supplementary Figure 11). Furthermore, we performed atomic-resolution HAADF-STEM and electron energy loss spectroscopy (EELS) mapping characterization. As shown in Fig. 3h–j, the atomic-solution HAADF-STEM images clearly demonstrate the well crystalized single nanocrystals. EELS analysis of a randomly selected region in a single nanocrystal confirmed the coexistence of Ru and Cr atoms. The corresponding EELS elemental mapping with subnanometer resolution (Fig. 3k) also shows a uniform uncorrelated spatial distribution of Cr, Ru, and O.

### OER catalytic performance in strong acidic media

**Fig. 4:**
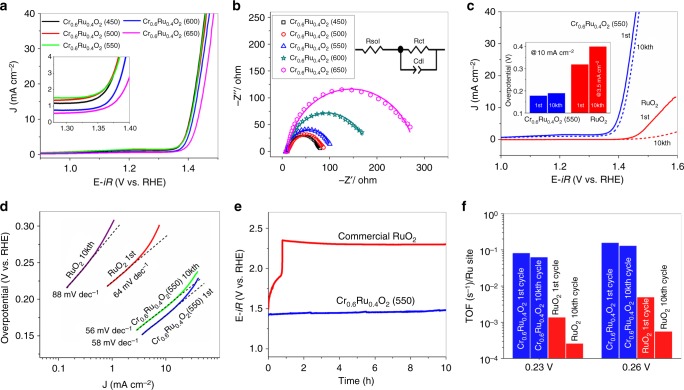
OER activity of Cr_0.6_Ru_0.4_O_2_ annealed at different temperature. **a** Electrocatalytic OER activities of Cr_0.6_Ru_0.4_O_2_ (450–650) nanoparticiles; **b** Nyquist plots at 1.395 V. Solid curves are the fitting results by using the equivalent circuit shown in the inset; **c** LSVs of Cr_0.6_Ru_0.4_O_2_ (550) and commerical RuO_2_ for the first and 10,000th cycle. Inset shows the comprarsion of overpotentials for Cr_0.6_Ru_0.4_O_2_ (550) and RuO_2_ at the current density of 10 mA cm^−2^ at the first and 10,000th cycle. For RuO_2_ after 10,000 cycles, the overpotential is corresponded to 3.5 mA cm^−2^ which is the maxium current density of its LSV curve; **d** Tafel plots of Cr_0.6_Ru_0.4_O_2_ (550) and RuO_2_ at first and 10,000th cycle; **e** Chronopotentiometry performance under constant current density of 10 mA cm^−2^ up to 10 h. **f** TOF results of Cr_0.6_Ru_0.4_O_2_ (550) and RuO_2_ at the first and 10,000th cycle

The OER activity of Cr_0.6_Ru_0.4_O_2_ powders annealed at different temperatures was studied in a strong acidic media (0.5 M H_2_SO_4_). The Cr_0.6_Ru_0.4_O_2_ based electrodes were prepared by drop-casting a water/ethanol and Nafion-based ink of Cr_0.6_Ru_0.4_O_2_ on the glassy carbon disk (see more details in methods section). Figure 4a shows the linear sweep voltammetry (LSV) results, where the rising current indicates the region where oxygen evolution occurred. Cr_0.6_Ru_0.4_O_2_ (450), Cr_0.6_Ru_0.4_O_2_ (500) and Cr_0.6_Ru_0.4_O_2_ (550) exhibit excellent initial OER activities, with onset potential of ~1.33 V vs. RHE, which represents an overpotential of ~100 mV. In addition, according to the suggested benchmark criteria^33^, Cr_0.6_Ru_0.4_O_2_ (450), Cr_0.6_Ru_0.4_O_2_ (500), and Cr_0.6_Ru_0.4_O_2_ (550) exhibited overpotentials of 175, 178 and 178 mV at the current density of 10 mA cm^−2^, respectively. Cr_0.6_Ru_0.4_O_2_ (600) and Cr_0.6_Ru_0.4_O_2_ (650) show slightly higher OER overpotentials (186 and 200 mV at 10 mA cm^−2^, respectively), but still lower than those reported in literatures^23,25^. Note that there is little difference in the PXRD patterns for Cr_0.6_Ru_0.4_O_2_ electrocatalysts annealed above 500 °C, the slightly lower OER activity for Cr_0.6_Ru_0.4_O_2_ (600) and Cr_0.6_Ru_0.4_O_2_ (650) might be ascribed to the lattice strain, which was also observed on IrO_2_^34^. Electrochemical impedance spectroscopy (EIS) measurement was further employed to reveal the catalytic property during OER. As shown in Fig. 4b, all the EIS spectra (Nyquist plots) at 1.395 V display a depressed semicircle, suggesting a charge-transfer process during the OER. These Nyquist plots can be well fitted by a simple equivalent electrical circuit which is composed of three components: solution resistance (*R*_sol_), charge transfer resistance (*R*_ct_), and double layer capacitance (*C*_dl_)^35–37^. The charge transfer resistance of Cr_0.6_Ru_0.4_O_2_ electrocatalyst generally increases with the applied annealing temperature. The larger charge transfer resistance for Cr_0.6_Ru_0.4_O_2_ electrocatalyst at higher annealing temperature can also be attributed to the lattice strain effects^34^. For Cr_0.6_Ru_0.4_O_2_ (550), the charge transfer resistance is 97.2 Ω, which is much smaller than that of commercial RuO_2_ (>4000 Ω) tested under the same conditions (Supplementary Figure 12, Supplementary Table 3), demonstrating a much faster kinetics for OER. Here the high Rct of commercial RuO_2_ is due to the fact that the OER reactions on RuO_2_ catalyst do not occur at 1.395 V. We thus measured the EIS spectra of RuO_2_ at higher voltages (Supplementary Figure 13). It shows that the Rct of RuO_2_ dramatically decreased with the increasing voltage applied. At 1.55 V, the Rct is 45.6 Ω, and the corresponding area-specific Rct is 3.2 Ω cm^2^, comparable to those of literatures reported^38^. In addition, we also measured the EIS spectra of Cr_0.6_Ru_0.4_O_2_ (550) at higher voltages (Supplementary Figure 14). The results show that Cr_0.6_Ru_0.4_O_2_ (550) has rather small Rct, with a value of 10.6 Ω at 1.47 V, corresponding to the area-specific Rct value of 0.7 Ω cm^2^. The durability of Cr_0.6_Ru_0.4_O_2_ electrocatalysts were assessed by cycling the catalysts between 1.2 and 1.6 V at a sweep rate of 100 mV s^−1^ in 0.5 M H_2_SO_4_ for 10,000 cycles. For Cr_0.6_Ru_0.4_O_2_ electrocatalyst annealed at 450 °C, the overpotential (at 10 mA cm^−2^) dramatically decreased from 177 mV at the first cycle to 242 mV at the 10,000th cycle (Supplementary Figure 15) due to the relatively unstable structures under the acidic solutions. In contrast, Cr_0.6_Ru_0.4_O_2_ electrocatalyst annealed above 500 °C exhibited stable OER performance, with slight overpotential decrease (<20 mV at 10 mA cm^−2^) after 10,000 cycles (Supplementary Figure 15). Notably, Cr_0.6_Ru_0.4_O_2_ (550) showed only 11 mV overpotential decrease (at 10 mA cm^−2^) after 10,000 cycles (Fig. 4c). The high stability of Cr_0.6_Ru_0.4_O_2_ (550) was also confirmed by TEM images of Cr_0.6_Ru_0.4_O_2_ (550) after 10,000 cycles, where no crystallinity or morphology change was observed (Supplementary Figure 16). In addition, the ICP-MS experiments (Supplementary Table 4) show that less than 2.5% Ru and 8% Cr of Cr_0.6_Ru_0.4_O_2_ (550) were dissolved in the acidic electrolyte solution after 10,000 cycles, which results in the slight degradation of OER performance. Note that such leaching content is smaller than those of recently reported excellent OER catalysts for acidic condition^23,27^.

In terms of both activity and stability, Cr_0.6_Ru_0.4_O_2_ (550) represents the best-performance OER electrocatalyst among the Cr_0.6_Ru_0.4_O_2_ electrocatalysts annealed between 450 and 650 °C, with overpotential (at 10 mA cm^−2^) of 178 mV at the first cycle and 189 mV at the 10,000th cycle. For further comparison, the OER performance of commercial RuO_2_ powder with particle size of ~30 nm (Supplementary Figure 17) was also tested under the same conditions. As shown in Fig. 4c, RuO_2_ exhibited much lower activity and stability compared to Cr_0.6_Ru_0.4_O_2_ (550). The overpotential at 1 and 10 mA cm^−2^ of RuO_2_ were measured to be 240 mV and 297 mV, respectively, which are consistent with those reported in literatures (Supplementary Table 5)^38^. After 10,000 cycles, the OER activity was dramatically decreased and became even negligible compared to the initial cycle. Figure 4d shows the Tafel plots of Cr_0.6_Ru_0.4_O_2_ (550) and RuO_2_ at the first and 10,000th cycle. The Tafel slope for RuO_2_ dramatically rose from 64 mV dec^−1^ to 88 mV dec^−1^ after 10,000 cycles. In contrast, the Tafel slope for Cr_0.6_Ru_0.4_O_2_ (550) slightly decreased from the initial value of 58 mV dec^−1^ to 56 mV dec^−1^ after 10,000 cycles. To further confirm the difference on stability of Cr_0.6_Ru_0.4_O_2_ (550) and RuO_2_ in catalytic performance, chronopotentiometry was examined under a constant current density. According to the suggested benchmark criteria in previous reports^23,33^, a current density of 10 mA cm^−2^ was used in the present study. Figure 4e shows the corresponding potential change for both Cr_0.6_Ru_0.4_O_2_ (550) and RuO_2_. The potential for RuO_2_ electrocatalyst changed from 1.5 to 1.9 V in 40 min and rose sharply to 2.19 V, essentially losing all its activity. On the contrary, the Cr_0.6_Ru_0.4_O_2_ (550) electrocatalyst remained essentially stable throughout the 10 h chronopotentiometry test. Furthermore, the turnover frequency **(**TOF) of Cr_0.6_Ru_0.4_O_2_ (550) and RuO_2_ was also calculated by dividing the number of oxygen molecules generated by the number of Ru sites under an assumed 100% Faradaic efficiency (Fig. 4f)^39^. Cr_0.6_Ru_0.4_O_2_ (550) showed a TOF value of 0.15 s^−1^ at the overpotential of 260 mV for the first cycle and slightly decreased to 0.13 s^−1^ for the 10,000th cycle. However, under the same condition, the TOF of RuO_2_ was decreased by an order of magnitude, changing from 4.9 × 10^−3^ s^−1^ at the first cycle to 5.5 × 10^−4^ s^−1^ for the 10,000th cycle. The same TOF change trend was also observed at an overpotential of 230 mV. It should be noted that all the Ru atoms including inaccessible ones in bulk were treated as surface sites in this TOF calculation, which thus underestimated the true TOF values^40^. In addition, we further calculated the electrochemically active surface area (ECSA), roughness factor of Cr_0.6_Ru_0.4_O_2_ (550) and RuO_2_ electrode, and plotted the LSVs with respect to the ECSA (Supplementary Figure 18-20, Supplementary Table 6). The results show that the enhanced activity of OER performance of CrO_2_-RuO_2_ solid solution is not just enhanced by the ECSA, and the intrinsic activity arising from the Cr ions plays an more important role.

In short, Cr_0.6_Ru_0.4_O_2_ (550) exhibits superior OER performance compared to RuO_2_ catalysts or other RuO_2_-based catalysts reported to date. Notably, it even outperforms the IrO_2_-based catalysts, which represent the state-of-the-art electrocatalyst for OER in acidic media (Table 1). An exhaustive comparison with other reported OER catalysts in acidic media is shown in Supplementary Table 7. It shows that the mass activity of Cr_0.6_Ru_0.4_O_2_ (550) at 270 mV (229 A g^−1^) is also much higher than those reported in literatures. In addition, the OER performance of CrO_2_ powder was also measured as a reference. As expected, no OER activity was observed on CrO_2_ powder (Supplementary Figure 21), suggesting that the synergic effects of Ru(IV) and Cr (IV) components in Cr_0.6_Ru_0.4_O_2_ structure are responsible for the excellent OER performance.

**Table 1:** Selected catalysts with high OER performance

Catalysts	Substrate	Electrolyte	Overpotential at specific current density	Chronopotentiometry at specific current density	Ref.
RuO_2_	Ti	0.5 M H_2_SO_4_	240 mV@1 mA cm^−2^	–	^38^
IrO_2_	GCE	0.1 M H_4_ClO_4_	~430 mV @10 mA cm^−2^	–	^14^
BaYIrO_6_	Au	0.1 M H_4_ClO_4_	~315 mV @10 mA cm^−2^	1 h@10 mA cm^−2^	^23^
IrO_x_/SrIrO_3_	Cu wire	0.5 M H_2_SO_4_	270–290 mV @10 mA cm^−2^	30 h@10 mA cm^−2^	^25^
Y_2_Ru_2_O_7-δŸ_	GCE	0.1 M H_4_ClO_4_	270 mV @1 mA cm^−2^	8 h@ 1 mA cm^−2^	^27^
IrCoNi PHNCs	CFP	0.1 M HClO_4_	303@10 mA cm^−2^	3.3 h@5 mA cm^−2^	^67^
W_0.57_Ir_0.43_O_3-δ_	FTO	1 M H_2_SO_4_	370@10 mA cm^−2^	0.6 h@10 mA cm^−2^	^68^
IrNi NCs	CFP	0.1 M HClO_4_	280@10 mA cm^−2^	2 h@5 mA cm^−2^	^69^
Ir	GF	0.5 M H_2_SO_4_	290@10 mA cm^−2^	10 h@10 mA cm^−2^	^70^
Cr_0.6_Ru_0.4_O_2_ (550)	GCE	0.5 M H_2_SO_4_	178 mV @10 mA cm^−2^	10 h@ 10 mA cm^−2^	This work

**Fig. 5:**
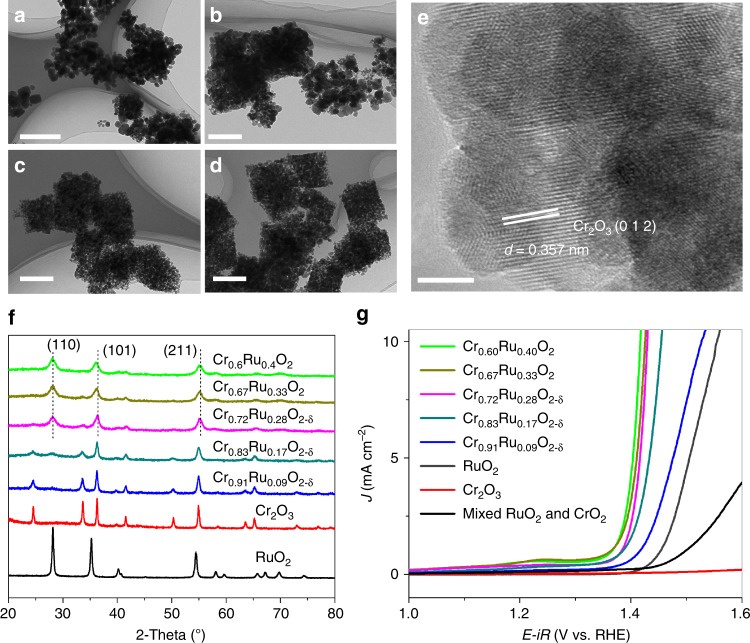
Evolution of chromium–ruthenium oxides with different Cr/Ru ratios: **a**–**d** TEM images of Cr_0.91_Ru_0.09_O_2-δ_, Cr_0.83_Ru_0.17_O_2-δ,_ Cr_0.72_Ru_0.28_O_2-δ_ and Cr_0.67_Ru_0.33_O_2_, respectively (scale bars, 200 nm); **e** HR-TEM image of Cr_0.83_Ru_0.17_O_2-δ_ (scale bar, 5 nm); **f** PXRD patterns, Cr_2_O_3_ powder was obtained from directly annealing pure MIL-101 (Cr); **g** LSV results

We further synthesized a series of Cr–Ru oxides with different Cr ratios to investigate the Cr role on the catalytic property. By varying the mass of RuCl_3_ in THF solution, we prepared MIL-101-RuCl_3_ precursors with different RuCl_3_ loading, and then obtained Cr–Ru oxides with Cr/Ru ratios varying from 9:1 to 6:4 after annealing (Cr/Ru ratios were determined by ICP-MS measurements). Figure 5 shows the corresponding morphology and structure evolution of Cr–Ru oxides. Directly annealing MIL-101(Cr) without loading RuCl_3_ at 450 °C, we only obtained Cr_2_O_3_ nanoparticles with high crystallinity (Supplementary Figure 22). After loading RuCl_3_ into MIL-101 (Cr), CrO_2_-RuO_2_ solid solution phase started to emerge after annealing. This is because RuO_2_ and CrO_2_ share the same rutile structure and have similar lattice constants, and the presence of Ru would induce the formation of RuO_2_–CrO_2_ solid solution. For Cr_0.91_Ru_0.09_O_2-δ_ and Cr_0.83_Ru_0.17_O_2-δ_ (*δ* was used to balance the valance of Cr^3+^ for the powders with mixed phase of Cr_2_O_3_ and CrO_2_–RuO_2_ solid solution) with low Ru content, the major phase is still Cr_2_O_3_, which can be clearly observed from the PXRD patterns in Fig. 5e. In contrast, for Cr_0.72_Ru_0.28_O_2-δ_ with higher Ru content, the CrO_2_–RuO_2_ solid solution turn to be the major phase, and for Cr_0.67_Ru_0.33_O_2_ and Cr_0.6_Ru_0.4_O_2_, pure phase of CrO_2_–RuO_2_ solid solution was formed. Note that, all the peaks shift slightly to the left side as the Ru content increases, which is a characteristic of RuO_2_–CrO_2_ solid solution. We further calculated the lattice parameters of Cr_1-x_Ru_x_O_2_ with solid solution as the major or pure phase (i.e., Cr_0.72_Ru_0.28_O_2-δ_, Cr_0.67_Ru_0.33_O_2_, and Cr_0.6_Ru_0.4_O_2_). As shown in Supplementary Figure 23, the c parameter varies nearly linearly with the composition. This quasi-linear relationship is in good agreement with the Vegard’s law. Although the shift of the *a* parameter shows the same trend as the c parameter when the Ru content increases, there is a deviation for the *a* parameter according to the Vegard’s law. This deviation was possibly due to the little difference of *a* parameter between RuO_2_ (*a* = 4.499 Å) and CrO_2_ (*a* = 4.421 Å), and/or the existence of some defects in the lattice along the *a* axis^41,42^. It is noteworthy that there is a pre-oxidation peak of the solid solution samples, which can be ascribed to the pre-oxidation of Cr. However, no such pre-oxidation peak was observed on Cr_2_O_3_ sample annealed at 450 °C, which can be attributed to its large crystal size (Supplementary Figure 24a) and relatively low active surface area that could cause low conductivity and activity. We thus prepared Cr_2_O_3_ with much smaller particle sizes (Supplementary Figures 22 and 24b) by annealing MIL-101(Cr) at lower temperature (300 °C). Indeed, herein we also observed this pre-oxidation peak on the Cr_2_O_3_ with less crystallinity (Supplementary Figure 25), albeit the peak was weak. The position of pre-oxidation peak of Cr_2_O_3_ was slightly higher than that of Cr_1-x_Ru_x_O_2_, which was possibly due to the synergistic effect of Cr and Ru in Cr_1-x_Ru_x_O_2_. Due to the saturation adsorption limit, we are unable to prepare Cr–Ru oxides with Cr/Ru ratio lower than 0.6/0.4. LSV results show that the OER performance of Cr–Ru oxides is highly correlated to the Ru/Cr ratio. Cr_0.91_Ru_0.09_O_2-δ_ and Cr_0.83_Ru_0.17_O_2-δ_ show moderate performance due to the high content of inactive Cr_2_O_3_ phase. With increasing Ru composition, the OER activity can be dramatically improved because the CrO_2_–RuO_2_ solid solution evolved as the major phase or even pure phase (Fig. 5f). However, it is noteworthy that the OER performance of Cr_0.91_Ru_0.09_O_2_ with small amount of CrO_2_-RuO_2_ solid solution phase is still higher than that of RuO_2_, highlighting the crucial role of Cr ions on the improved activity towards OER. We also measured the OER performance of mixed RuO_2_ and CrO_2_ sample. The result shows that mixed RuO_2_ and CrO_2_ has very poor OER performance, even much lower than that of pristine RuO_2_. Note that the conductivity plays an important role in the OER process, and it might not be a good comparison to the chromium-ruthenium oxides if some residual carbon species inherent from MOF precursor exist in our samples. Therefore, we further preformed Raman and thermogravimetric (TG) measurement of the samples to detect the residual carbon. As shown in Supplementary Figures 26 and 27, no signal of the residual carbon can be observed. Nevertheless, we added carbonaceous additive (commercial acetylene black that has high conductivity) to the mixed CrO_2_–RuO_2_, denoted as mixed CrO_2_–RuO_2_/C. As shown in Supplementary Figure 28, the OER activity of mixed CrO_2_–RuO_2_/C was enhanced after the addition of carbon black, but still lower than that of pure RuO_2_, indicating the important synergistic effect of Cr^4+^ role as a participating lattice ion.

### Intrinsic mechanism for the excellent OER performance

**Fig. 6:**
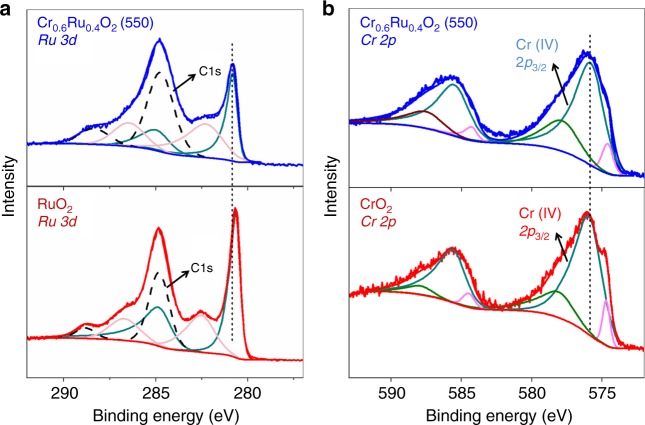
XPS of Cr_0.6_Ru_0.4_O_2_ (550) for Ru 3*d* and Cr 2*p*. **a** XPS of Cr_0.6_Ru_0.4_O_2_ (550) and RuO_2_ for Ru 3*d* regions. **b** XPS of Cr_0.6_Ru_0.4_O_2_ (550) and CrO_2_ for Cr 2*p* regions. The blue and red smoothing lines are fitting results of the sum of individual components. For Ru 3*d*, color codes are used to distinguish the different spin-orbit components, dark cyan for primary Ru 3*d*_3/2_ and 3*d*_5/2_ spin states, and light magenta for satellite Ru 3*d*_3/2_ and 3*d*_5/2_ spin states

We first performed X-ray photoelectron spectroscopy (XPS) to access the surface chemical state of Cr_0.6_Ru_0.4_O_2_ (550). As shown in Fig. 6a, there are two sets of doublet peaks for Ru 3*d* in the region between 280 and 290 eV, corresponding to the doublet peaks for Ru (IV) 3*d*_5/2_, 3*d*_3/2_ and their satellite peaks^43^. The primary Ru 3*d*_5/2_ and 3*d*_3/2_ peaks of RuO_2_ centered at 280.6 and 284.8 eV, respectively, which are consistent with literatures^44,45^. A shift to higher binding energy can be clearly observed on Cr_0.6_Ru_0.4_O_2_ (550) compared to RuO_2_, suggesting a lower electron density at the Ru sites. This can be attributed to the electron withdrawing effect of Cr (IV) in the lattice. Note that the observed C1s peaks are resulting from the carbon adhesive tape used in XPS measurement and environmental corrosion carbon. Indeed, as mentioned above, Raman characterization and thermogravimetric analysis confirmed that there is negligible carbon component in the Cr_0.6_Ru_0.4_O_2_ catalysts (Supplementary Figures 26 and 27). For Cr 2*p*, three sets of doublet peaks can be observed on Cr_0.6_Ru_0.4_O_2_ (550) and CrO_2_ in the region between 570 and 595 eV (Fig. 6b). The primary peaks at ~576.0 eV correspond to Cr (IV) 2*p*_3/2_^46,47^. For Cr (IV) 2*p*_3/2_ of Cr_0.6_Ru_0.4_O_2_ (550), a shift to lower binding energy is observed compared to CrO_2_, implying a higher electron density at Cr sites, which confirms the withdrawing effect of Cr (IV) in Cr_0.6_Ru_0.4_O_2_ (550). For the other two peaks in the Cr 2*p*_3/2_ region, the smaller ones at ~575.0 eV can be assigned to Cr (III) 2*p*_3/2_, which implies the appearance of a small amount of Cr (III) sites on the outer surface of Cr_0.6_Ru_0.4_O_2_ (550) and CrO_2_ crystals^47,48^, and the larger ones at ~577.9 eV can be assigned to CrO_2_H, which likely resulted from the reaction between Cr (IV) and the proton from environment^49^. Additional XPS spectra for wide scan and Ru 3*p* regions are shown in Supplementary Figures 29 and 30.

**Fig. 7:**
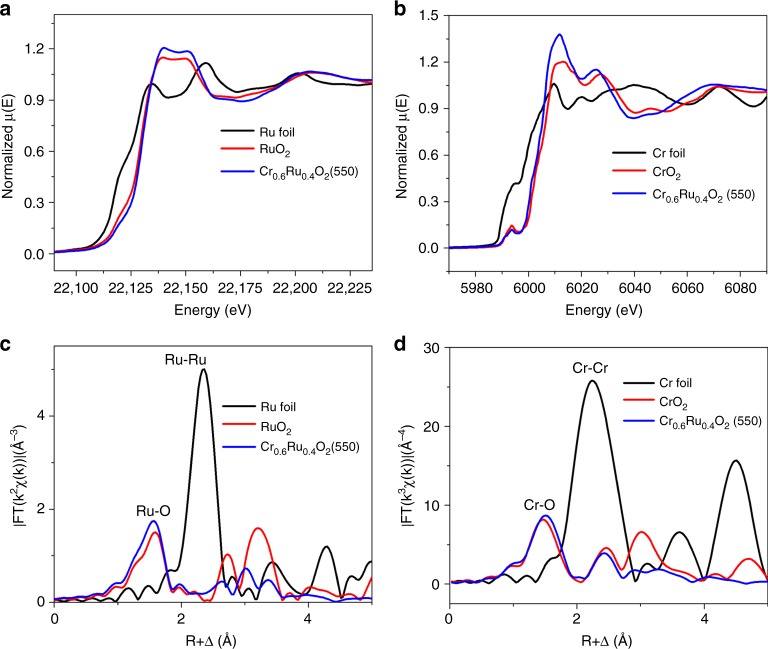
XAS analysis of Cr_0.6_Ru_0.4_O_2_ (550) electrocatalyst. **a** Normalized Ru K-edge XANES spectra of Cr_0.6_Ru_0.4_O_2_ (550), Ru foil and commercial RuO_2_. **b** Normalized Cr K-edge XANES spectra of Cr_0.6_Ru_0.4_O_2_ (550), Cr foil, and commercial CrO_2_. **c** Fourier transformed EXAFS spectra of Ru edge for Cr_0.6_Ru_0.4_O_2_ (550), Ru foil and commercial RuO_2_. **d** Fourier transformed EXAFS spectra of Cr edge for Cr_0.6_Ru_0.4_O_2_ (550), Cr foil, and commercial CrO_2_

To elucidate the atomic structure of Cr_0.6_Ru_0.4_O_2_(550), X-ray absorption spectroscopy (XAS) characterization was further employed. Figure 7a shows the X-ray absorption near edge structure (XANES) of Ru K-edge region of the rutile-type Cr_0.6_Ru_0.4_O_2_(550). Pure Ru metal foil and RuO_2_ powder were also measured as reference. The shoulder near the adsorption threshold of Ru foil is corresponding to the 1*s* to 4*d* transition. For RuO_2_ and Cr_0.6_Ru_0.4_O_2_(550), this shoulder is weaker, because the increased lattice symmetry prevents the mixing of 4*d* and 5*p* orbitals. The observed transition energy of XANES (corresponding to the 1*s* to 5*p* transition) for RuO_2_ and Cr_0.6_Ru_0.4_O_2_ is higher than that for Ru. This can be attributed to the formation of Ru–O bonds, which pushes up the empty state of 5*p* oribials of Ru atoms^50^. We further analyzed the absorption energy (*E*_0_, determined from the first maximum in the first-order derivative), which is proportional to the oxidation state of transition metals^51,52^. We found that the absorption energy for Cr_0.6_Ru_0.4_O_2_(550) (*E*_0_ = 22129.9 eV) was similar with the value of RuO_2_ (*E*_0_ = 22129.5 eV), implying that the oxidation state of Ru in Cr_0.6_Ru_0.4_O_2_(550) is close to +4. The slightly higher absorption energy can be attributed to the electron withdrawing effect of the neighboring lattice Cr^4+^ ion, consistent with the XPS analysis results. Ru K-edge extended X-ray absorption fine structure (EXAFS) analysis was used to reveal the initial information on the Ru−O and Ru−Ru bonds. The corresponding Fourier transformed (FT) radial structure based on the *k*^2^-weighted EXAFS is displayed in Fig. 7c. The peak at 1.59 Å for RuO_2_ is associated with the back scattering of Ru–O in the first shell^27^. In contrast, the Ru–O bond length in Cr_0.6_Ru_0.4_O_2_(550) is slightly shortened to 1.55 Å, in line with the slightly higher absorption energy for Cr_0.6_Ru_0.4_O_2_(550). The peaks at 2.73 and 3.21 Å for RuO_2_ arise from the back scatterings of Ru−Ru in the second and third shell^53^. These peaks for Cr_0.6_Ru_0.4_O_2_(550) are assigned to the back scatterings Ru–Ru and Ru–Cr. The decreased intensity (i.e., vibrational amplitude) should be ascribed to the extremely small particle size (less than 15 nm)^54,55^. Furthermore, these peaks are also shifted to the left. Clearly, the presence of Cr can profoundly alter the local electronic structures of Ru and the associated Ru-O bonding, which directly determine the OER activity. Accordingly, Cr K-edge XANES and EXAFS were also used to examine the Cr oxide state, and Cr–O bond in Cr_0.6_Ru_0.4_O_2_(550) (Fig. 7b, d). The absorption energy of Cr_0.6_Ru_0.4_O_2_(550) (*E*_0_ = 6006.7 eV) is higher than that of Cr metal (*E*_0_ = 5989.0 eV), but close to the value of CrO_2_ (*E*_0_ = 6006.8 eV). In addition, the peak in the region of pre-edge absorption is also a characteristic of the formation of Cr^4+^, corresponding to the 1*s* to 3*d* transition^56^. The slightly lower absorption energy can be attributed to the electron withdrawing effect of Cr^4+^ ion, in agreement with the XPS results and Ru oxidation analysis. In addition, as shown in Fig. 7d, the Cr–O length for CrO_2_ is 1.47 Å. In Cr_0.6_Ru_0.4_O_2_(550), the Cr–O is slightly elongated to 1.50 Å, in accordance with the EXAFS result of Ru K-edge. For comparison, we also measured the Cr K-edge XANES of Cr_0.6_Ru_0.4_O_2_ (450). As shown in Supplementary Figure 31, the intensity of the pre-edge peak of Cr_0.6_Ru_0.4_O_2_(450) is above that of Cr_0.6_Ru_0.4_O_2_(550), indicating a lower symmetry environment of the Cr atoms in Cr_0.6_Ru_0.4_O_2_(450)^56^. It confirmed that the fine structure of Cr_0.6_Ru_0.4_O_2_(450) is different from that of Cr_0.6_Ru_0.4_O_2_(550), i.e., the pure phase rutile Cr–Ru oxide has not been well formed.

**Fig. 8:**
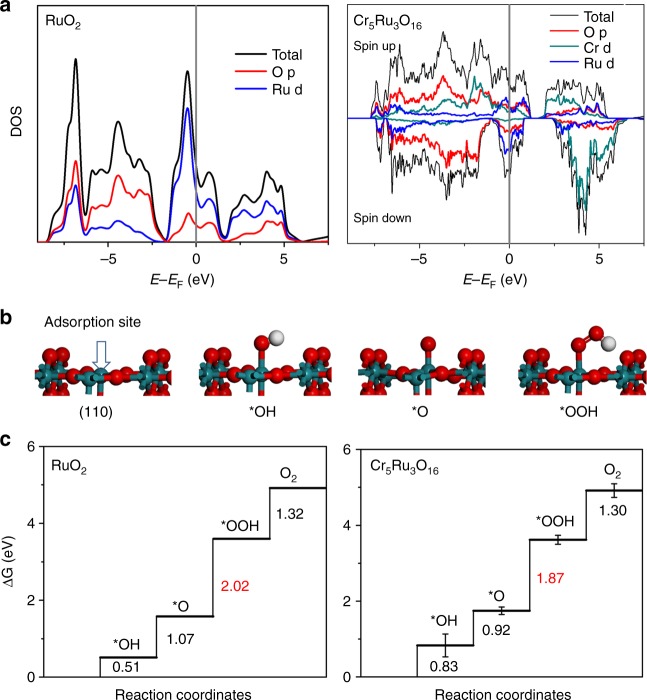
DFT calculations on the studied CrO_2_-RuO_2_ solid solution system. **a** DOS of RuO_2_ and Cr_5_Ru_3_O_16_; **b** The four-step OER process; **c** The calculated free energy diagrams for RuO_2_ and Cr_5_Ru_3_O_16_

Finally, we carried out DFT calculations in order to understand the promoted OER performance of CrO_2_–RuO_2_ electrocatalyst. Here we constructed a simulation model of Cr_5_Ru_3_O_16_, which has a composition close to the experimentally measured value. We assumed that Cr and Ru are distributed as evenly as possible in the rutile-like crystal. The simulated PXRD pattern of the relaxed structure was in good agreement with experiments. Based on the Bader charge analysis, the partial charge of Ru in bulk RuO_2_ was calculated to be  +1.73|e|. For comparison, the Ru cation in Cr_5_Ru_3_O_16_ possesses a higher positive charge of +1.92|e|. Accordingly, the partial charge on the neighboring Cr cation decreases from+1.89|e| in CrO_2_ to +1.84|e| in Cr_5_Ru_3_O_16_. Clearly, the electron transfer from Ru to Cr is consistent with the XANES results. More positively charged Ru cations lead to smaller cation radius, and the corresponding peaks in EXAFS slightly shift to the left. Moreover, the highly oxidized state of Ru implies the improved ability for the oxidation of water to oxygen, namely OER. We further plotted the density of states (DOS) to discern the nature of the electronic structures. As shown in Fig. 8a, the incorporation of Cr apparently altered the DOS of nonmagnetic RuO_2_. The occupation at the Fermi level decreases from 2.01 states/(eV*cell*spin) in RuO_2_ to 1.07 states/(eV*cell*spin) in the solid solution, indicating the stabilization of crystalline structure^57^. Owing to the localized nature of O *p*-band, its band center is known as an effective descriptor to predict intrinsic OER activity of oxides^58^. Considering the less electron number of Cr^4+^ (2e^−^) than that of Ru^4+^ (4e^−^), the Fermi level of solid solution is shifted downward due to the band filling effect. Correspondingly, the O *p-*band center moves closer to the Fermi level in Cr_5_Ru_3_O_16_ (−2.48 eV), compared with the value of −2.91 eV in RuO_2_. Clearly, the upshift O *p-*band suggests the enhanced activity for OER. The detailed projected DOS of Cr_5_Ru_3_O_16_ are displayed in Supplementary Figures 32-33, also consistent with previous theoretical study on CrO_2_–RuO_2_ structures^59^. The oxygen 2*p* (O-*p*) states below ~1.5 eV overlap with part of the metal *d-*bands. Metal t_2g_ orbitals show a unique spread and strong peak at the edge of valence band, especially in Ru *d*-orbitals. Interestingly, the O-*p* orbital (the major component is below −2 eV) and Ru-*d* bands at higher energy state are well separated in RuO_2_ (Fig. 8a). In contrast, the relatively low energy Cr t_2g_ orbitals can enhance the hybridization of O-*p* orbital, thus further push O *p*-center closer to Fermi level in the solid solution. Note that the empty *e*_g_ orbitals of Cr intensely strengthen the DOS ranging from about 2 to 6 eV and the increased DOS related to σ antibonding state suggests a weak Cr–O binding strength.

On the other hand, we also calculated the free energy profiles of OER to directly compare the OER activities of RuO_2_ and CrO_2_–RuO_2_ solid solution. A slab model containing 32 O and 16 metal atoms was employed, in which the Cr/Ru ratio was kept as 5:3. Here we considered a four-step OER mechanism with CHE model to provide a general view^60–62^. We first focused on the (110) facet as the surface model, because it has been identified as the most stable from calculation of surface energy (Supplementary Table 8). We constructed surfaces of both RuO_2_ and solid solution for comparison. Five different configurations of solid solution surface were further modeled to average the calculated energy barriers. As shown in Fig. 8b, the five-coordinated surface Ru was identified as the reactive adsorption site. Interestingly, under the oxidation condition in water, the Ru site could readily adsorb OH to form *OH. For all the models, the formation of *OOH was found to be the rate determining step (RDS). On the Cr_5_Ru_3_O_16_ surface, the free energy change of RDS at the Ru site was calculated to be 1.87 eV, which is approximately 0.1 eV lower than that on RuO_2_ surface (2.02 eV) (Fig. 8c). It is consistent with the decreased overpotential of 100 mV measured in experiments. To further corroborate the synergistic effect of Cr ions on the enhanced OER activity, we considered two more cases with different Ru concentration and structures, as shown in Supplementary Figure 34. In the first one, there are only surface Ru and sub-surface Cr ions, in which the energy barrier for RDS was calculated to be 1.81 eV. In the second case, one Cr cation on CrO_2_(110) surface was replaced by Ru, yielding a reduced RDS barrier of 1.75 eV, which is lower than that of original RuO2 surface. On the other hand, we also investigated two relatively less stable surfaces, namely (200) and (101) facets, which were observed in HR-TEM image (Fig. 3d). In a previous experimental study^63^, these two surfaces of RuO_2_ were found to be more active. Our DFT results (Supplementary Figure 35) are consistent with this work, and the corresponding energy barriers of RDS on (200) and (101) surfaces were calculated to be 1.79 and 1.96 eV, respectively. In CrO_2_–RuO_2_ system, these two barriers were further decreased by 0.04 and 0.02 eV, respectively.

## Discussion

In summary, by using Cr-based MOF, we have developed a cost effective rutile Cr_0.6_Ru_0.4_O_2_ electrocatalyst with superior OER activity and stability in acidic media. Our experimental results and DFT calculations revealed the profound influence of Cr on the OER performance. The enhanced stability is related to the lower occupation at the Fermi level, while the higher activity results from the altered electronic structures. The calculated free energy diagrams for OER further demonstrates a lower energy barrier for the formation of *OOH, which is the RDS. On the other hand, Ru plays the key role to induce the formation of ruile-structured CrO_2_ and thus CrO_2_–RuO_2_ solid solutions because RuO_2_ and CrO_2_ share the same rutile structure and have similar lattice constants. Note that direct annealing of the MIL-101(Cr) precursor only led to an inactive product of Cr_2_O_3_. These findings and results open a route to design highly active, stable and relatively low-cost electrocatalysts for OER in acidic media. To shed light on the further optimization, we have investigated and screened a series of possible rutile-like MO_2_–RuO_2_ systems, in which M is a tetravalent cation. In light of the altered electronic structures in CrO_2_–RuO_2_, we propose that the electron withdrawing on Ru ions can facilitate water oxidation and oxygen evolution. The calculated parameters of the cells and partial charges on Ru are summarized in Supplementary Table 9. It is noteworthy that the partial charge on Ru ions in CrO_2_–RuO_2_ is found to be the most positive. Interestingly, we found that MnO_2_ can also form solid solution with RuO_2_ and possess good OER performance, owing to the similar cell parameter with that of RuO_2_ and the highly positive partial charge on Ru (1.88 e). In contrast, we found that the reductive tetravalent cations, such as Nb and W, lead to electron accumulation on Ru. As a result, their corresponding solid solutions are expected to have lower OER activity. In addition to the formation of solid solution, we also anticipate that doping RuO_2_ with highly oxidizing metal ions, such as Ce^4+^, is another viable strategy to improve the OER activity. On the other hand, our preparation method for chromium ruthenium oxides solid solution electrocatalyst can be extended to prepare other rutile-structured electrocatalysts, such as the potentially active manganese ruthenium oxide and vanadium ruthenium oxide, or even non-Pt-group metal materials, such as chromium manganese oxide.

## Methods

### Materials

All chemicals were obtained from commercial suppliers at analytical grade and used as received without further purification. The commercial RuO_2_ and CrO_2_ were purchased from Sigma-Aldrich.

### MIL-101(Cr) synthesis

MIL-101(Cr) was prepared by a hydrothermal reaction following the procedure reported in our previously work^30^. The prepared MIL-101 (Cr) was activated at 150 °C under vacuum for 12 h for future use.

### Preparation of RuCl_3_-MIL-101 (Cr)

A series of RuCl_3_-MIL-101 (Cr) with different RuCl_3_ loading were prepared by mixing the desired amount of MIL-101 (Cr) and RuCl_3_ in THF solution. Typically, 0.2 g RuCl_3_ was dissolved in 30 ml tetrahydrofuran (THF) under stirring for 5 min. After that, 0.2 g MIL-101 (Cr) was slowly added into the RuCl_3_ solution under stirring. To ensure the uniform distribution of RuCl_3_ within the pores and thus the formation of homogeneous Cr–Ru oxide solid solution phase upon heating, the resulting mixture was further kept stirring at room temperature for 18 h to allow the compete loading of RuCl_3_ into MIL-101 (Cr) pores. After impregnation, the product was recovered by centrifugation, and washed four times with THF to remove RuCl_3_ remained on the outer surface of MIL-101 (Cr) particles. Finally, the resulting RuCl_3_-MIL-101 (Cr) was dried at 80 °C in air for 6 h. For the other RuCl_3_-MIL-101 (Cr) with lower RuCl_3_ loading, the amount of RuCl_3_ used was decreased to 0.15 g, 0.1 g, 0.05 g, and 0.025 g, respectively.

### Preparation of Cr_1-x_Ru_x_O_2_

Fifty microgram of RuCl_3_-MIL-101 (Cr) powder was placed in muffle furnace and heated to *T* (*T* = 450, 500, 550, 600, 650 °C) at a heating rate of 5 °C/min and held for 4 h. After cooling down to room temperatures, the resulting black products were collected, and denoted as Cr_1-x_Ru_x_O_2_ (T).

### Electrochemical measurements

In a typical procedure, 4 mg of Cr_1-x_Ru_x_O_2_ was added to 1 ml of water/ethanol (3:1, v/v) containing 15 µl Nafion aqueous solution (5%, Sigma-Aldrich), and dispersed by sonication for 30 min to generate a homogeneous black ink. Five microliter of the catalyst ink was drop-cast on a glassy carbon electrode (surface area: 0.07065 cm^2^) and dried in air at room temperature to form a thin film working electrode. For the mixed CrO_2_-RuO_2_/C ink preparation, 4 mg carbon black (commercial acetylene black that has high conductivity) was added. A three-electrode cell was employed to measure the OER electrochemical performance. The cell contained the glassy carbon working electrode, a counter electrode made of platinum wire (diameter: 0.5 nm), and a saturated Hg/Hg_2_SO_4_ reference electrode. All measurements were performed in 0.5 M H_2_SO_4_ acidic solution after purging with O_2_ (99.999%) for at least 30 min. The Hg/Hg_2_SO_4_ reference electrode was calibrated with a Pt wire electrode in H_2_-saturated 0.5 M H_2_SO_4_ solution. The potential difference between the Hg/Hg_2_SO_4_ reference electrode and reversible hydrogen electrode is 0.645 V. Cyclic voltammograms (CVs) tests were collected at a scan rate of 100 mV/s typically between 1.2 and 1.6 V. Linear sweep voltammetry (LSV) curves were recorded at a scan rate of 5 mV/s typically between 0.8 and 1.6 V. Chronopotentiometric measurements were conducted by applying constant current (10 mA cm^−2^) for up to 10 h. Electrochemical impedance spectroscopy (EIS) were performed at 0.75 V. The EIS results were presented in the form of Nyquist plot and fitted using ZView software with a representative equivalent electrical circuit.

### Electrochemically active surface area (ECSAs)

The ECSAs were estimated from the electrochemical double-layer capacitance of the catalytic surface. The double layer capacitance (*C*_DL_) was determined by measuring the non-Faradaic capacitive current charging from the scan-rate dependence of CVs. The potential window of CVs was 1.21–1.31 V vs. RHE (0.1 V potential window centered at the open-circuit potential of the system). The *C*_DL_ was given by the following equation:

1 $$i_{\mathrm{c}}{\mathrm{ = \nu C}}_{{\mathrm{DL}}},$$

where *ν* is the scan rate. The slope of the plot of *i*_c_ as a function of *ν* is equal to *C*_DL_.

The ECSA is calculated from the double layer capacitance according to:

2 $${\mathrm{ECSA = }}C_{{\mathrm{DL}}}{\mathrm{/}}C_{\mathrm{s}},$$

where *C*_s_ is the specific capacitance of the sample. We use general specific capacitances of *C*_s_ = 0.035 mF cm^−2^ based on typical reported values. The roughness factor (RF) is then calculated by dividing ECSA by 0.07065 cm^2^, the geometric area of the electrode.

### Material characterization

Power X-ray diffractions (PXRD) patterns of the samples were collected on a D8-Advance Bruker AXS diffractometer with Cu_kα_ (*λ* = 1.5418 Å) radiation at room temperature. In order to obtain high quality data for Cr_0.6_Ru_0.4_O_2_ (550), a very slow scan measurement was performed with a scan interval of 0.005° per step and a scan rate of 3 s per step. Structure analysis was conducted on Jade 2004. The lattice parameters were refined using GSAS software [A. C. Larson and R.B. von Dreele, Los Alamos, 1994]. Inductively coupled plasma-mass spectroscopy (ICP-MS) measurements were carried on NexION 300 (Perkin-Elmer). For the leaching measurements, the loading amounts of catalysts varied from 20 to 60 μg and the volume of the electrolyte was 100 ml. After 10,000 cycles, the electrolyte was concentrated to a final volume of ∼10 ml for ICP-MS analysis. The samples morphologies were examined using a field emission scanning electron microscope (SEM) (Hitachi, S-4800). SEM specimens were prepared by depositing sample powders on carbon adhesive tape on a SEM holder. Transmission electron microscopy (TEM) and high-resolution TEM (HR-TEM) images were recorded on Tecnai F20 microscope, and high-angle annular dark-field scanning transmission electron microscopy (HAADF-STEM) images were carefully recorded on Talos F200X and JEM-ARM200F. Atomic solution HAADF-STEM images were carefully recorded on JEM-ARM200F. For TEM specimen preparation, sample powders were firstly dispersed in ethanol by sonication, followed by dripping onto a carbon-coated copper grid. Nitrogen adsorption/desorption isotherms were measured on ASAP2020M apparatus at 77 K. The Brunauer−Emmett−Teller (BET) surface area was calculated over the range of relative pressures between 0.05 and 0.2. Before the measurements were performed, the samples were outgassed under vacuum at 160 °C for 12 h. X-ray photoelectron spectroscopy (XPS) spectra were recorded on the AXIS ULTRA using Al_Kα_ radiation. The X-ray absorption data (XAS) at the Ru-K edge and the Cr K edge of the samples, which were mixed with LiF to reach 50 mg, were recorded at room temperature in transmission mode using ion chambers using the BL14W1 beam line of the Shanghai Synchrotron Radiation Facility (SSRF), China. The station was operated with a Si (111) double crystal monochromator. The electron beam energy of the storage ring was 3.5 GeV and the maximum stored current was ~210 mA. The energy calibrations were performed using a Ru foil (22117 eV) or Cr foil (5989 eV). For Ru K-edge XAS, The extracted EXAFS signal, *χ*(k), was weighted by *k*^2^ in k-range from 3.8 to 15.6 Å^−1^ to obtain the magnitude. For Cr K-edge XAS, The extracted EXAFS signal, *χ*(k), was weighted by *k*^3^ in k-range from 3 to 12 Å^−1^ to obtain the magnitude.

### Turnover frequency calculation (TOF)

TOF was calculated based on the method reported in previous works^27,40^. This calculation assumes 100% Faradaic efficiency:

3 $${\mathrm{TOF = }}N_{{\mathrm{O2}}}{\mathrm{/}}N_{{\mathrm{Ru}}}$$

where *N*_O2_ is the number of O_2_ turnovers, calculated using the following formula:

4 $$\begin{array}{l}{{N}}_{{\mathrm{O2}}}{\mathrm{ = }}\left( {{\mathrm{j}}\,{\mathrm{mA}}\,{\mathrm{cm}}^{{\mathrm{ - 2}}}} \right) \times \left( {{\mathrm{A}}\,{\mathrm{cm}}^{\mathrm{2}}_{{\mathrm{oxide}}}} \right) \times \left( {{\mathrm{1}}\,{\mathrm{Cs}}^{{\mathrm{ - 1}}}{\mathrm{/1000}}\,{\mathrm{mA}}} \right) \times \\ \left( {{\mathrm{1}}\,{\mathrm{mol}}\,{\mathrm{e}}^ - {\mathrm{/96485}}\,{\mathrm{C}}} \right) \times \left( {{\mathrm{1}}\,{\mathrm{mol}}\,{\mathrm{O}}_{\mathrm{2}}{\mathrm{/4}}\,{\mathrm{mol}}\,{\mathrm{e}}^ - } \right) \times {{N}}_{\mathrm{A}} \cdot \end{array},$$

where *j* is the measured current density, *A* is the surface area of electrode, and *N*_A_ is Avogadro constant (6.02 × 10^23^ mol^−1^).

The number of Ru sites (N_Ru_) is calculated using the formula: (0.4 × (20 ×10^−6^ g) × *N*_A_/ molecular weight of Cr_0.6_Ru_0.4_O_2_) for Cr_0.6_Ru_0.4_O_2_, and ((20 × 10^−6^ g) × *N*_A_/molecular weight RuO_2_) for RuO_2_, respectively.

### Density functional theory (DFT) calculations

The DFT calculations were performed using the Vienna Ab-initio Simulation Package (VASP)^64^. The Perdew-Burke-Ernzerhof (PBE) functional of the generalized gradient approximation (GGA)^65^ was employed with projector augmented wave (PAW)^66^ method. The valence electronic configurations were O (2*s*, 2*p*), Ru (4*p*, 4*d*, 5*s*), Cr (3*p*, 3*d*, 4*s*), and H (1*s*). In particular, the *U*_eff_ of 3.7 eV was utilized for 3*d* orbital of Cr. Spin polarization was also considered. The energy cutoff for plane wave was set to 500 eV. The thresholds for electronic structure iteration and geometry relaxation were 10^−5^ eV and 0.03 eV/Å in force, respectively. Due to the conducting nature, the first order Methfessel-Paxton method with smearing of 0.1 eV was applied for optimization and tetrahedron method with Blöchl corrections was further used for the density of states (DOS) calculation. The lattices of RuO_2_ and Cr and Ru oxides solid solution were relaxed based on fixed rutile symmetry. 9 × 9 × 13 Monkhorst-Pack k-point grid was used to sample the Brillouin zone. Then four layered (110) facet was cleaved with the vacuum slab height of 20 Å. A 2 × 1 supercell containing 32 O and 16 metal atoms were studied with 5 × 5 × 1 Monkhorst-Pack k-point grid. To describe vdW interaction, empirical Grimme’s D3 correction was adopted.

The free energy of each species was calculated based on the following formula:

5 $$G = E_{{\mathrm{dft}}}+ E_{{\mathrm{zpe}}}{\mathrm{- T\Delta S}}$$

The zero-point energy and entropy correction were obtained from standard vibrational calculation, whereas the free energy of O_2_ was derived according to experimental standard formation energy of liquid water:

6 $${\mathrm{G}}\left( {{\mathrm{O}}_{\mathrm{2}}} \right){\mathrm{ = }}\,{\mathrm{4}}{\mathrm{.92}}\,{\mathrm{eV + 2G}}\left( {{\mathrm{H}}_{\mathrm{2}}{\mathrm{O}}} \right){\mathrm{ - 2G}}\left( {{\mathrm{H}}_{\mathrm{2}}} \right)$$

Moreover, we have also attempted to screen a series of potential solid solutions for further prediction, which are composed of RuO_2_ and other rutile-like oxides, including TiO_2_, VO_2_, CrO_2_, MnO_2_, GeO_2_, NbO_2_, MoO_2_, RhO_2_, SnO_2_, WO_2_, and PbO_2_. The cell sizes of the bulk models were allowed to relax in the calculations at the aforementioned level. The calculated theoretical lattice parameters are listed in Supplementary Table 9. Ideally, the closer cell parameters for the two MO_2_ crystals, the higher possibility the solid solution can be formed. Besides, the atomic charges on Ru atoms in these solid solution systems were calculated based on the Bader charge analysis. Here the higher positive partial charge compared with Ru in bulk RuO_2_ indicates that the Ru ion in solid solution would donate electrons to other metals, and accordingly its oxidizing ability is strengthened to promote OER performance. For comparison, the number of valence electron and electronegativity of various metals are labeled in Supplementary Table 9. However, it seems that they have trivial influence on the electronic distribution on Ru.

## Supplementary information

PDF version of Supplementary Information

Peer Review File

## Data Availability

The authors declare that all the published data supporting the findings of this study are available within the article and its supplementary information files.
