# Supplementary material for: Chromium-ruthenium oxide solid solution electrocatalyst for highly efficient oxygen evolution reaction in acidic media
Source: Nat Commun. 2019 Jan 11;10:162. doi: 10.1038/s41467-018-08144-3 (PMC6329788; doi:10.1038/s41467-018-08144-3)
Supplement: Supplementary file 1 — PDF version of Supplementary Information [file 41467_2018_8144_MOESM1_ESM.pdf]

Supplementary Information

Chromium-ruthenium oxide solid solution electrocatalyst for highly efficient oxygen evolution reaction in acidic media

Yichao Lin et al.

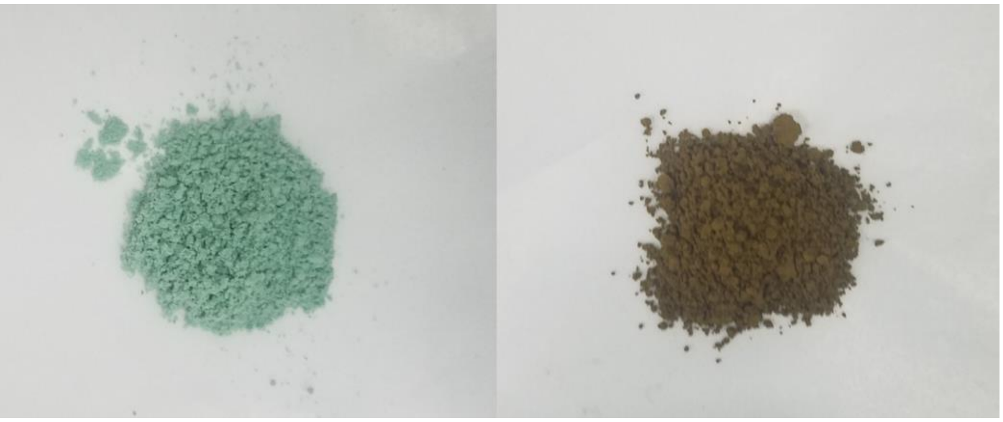

Supplementary Figure 1. Digital photographs of MIL-101(Cr) before (left) and after (right) loading $RuCl_3$.

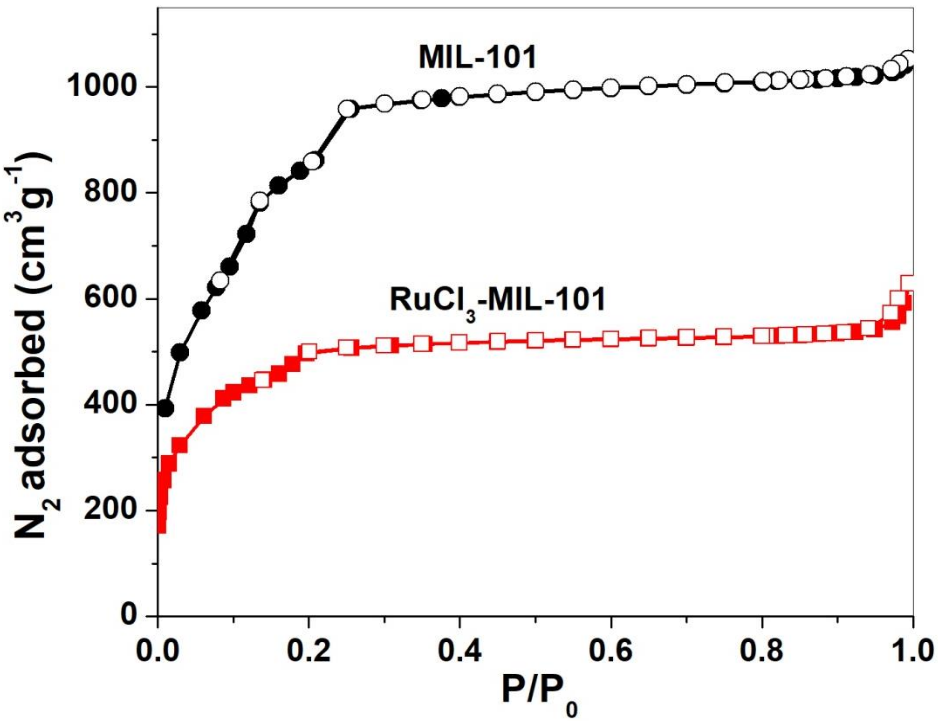

Supplementary Figure 2. N_2 adsorption/desorption isotherms of MIL-101 (Cr) and $RuCl_3$-MIL-101 (Cr). Filled, adsorption; Blank, desorption.

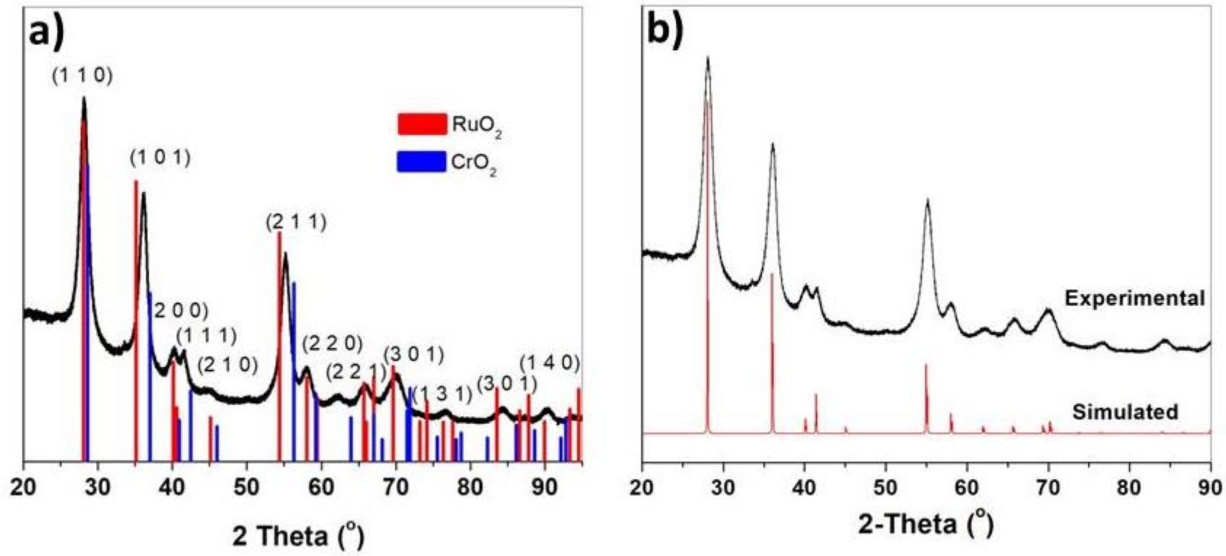

Supplementary Figure 3. Experimental PXRD data vs. (a) standard CrO_2 and RuO_2 (JCPDS No.09-0332 and 43-1027) and (b) simulated $Cr_{0.6}Ru_{0.4}O_2$ structure. The PXRD is acquired by a very slow scan with a scan step of 0.005^o and a scan rate of 4 second per step.

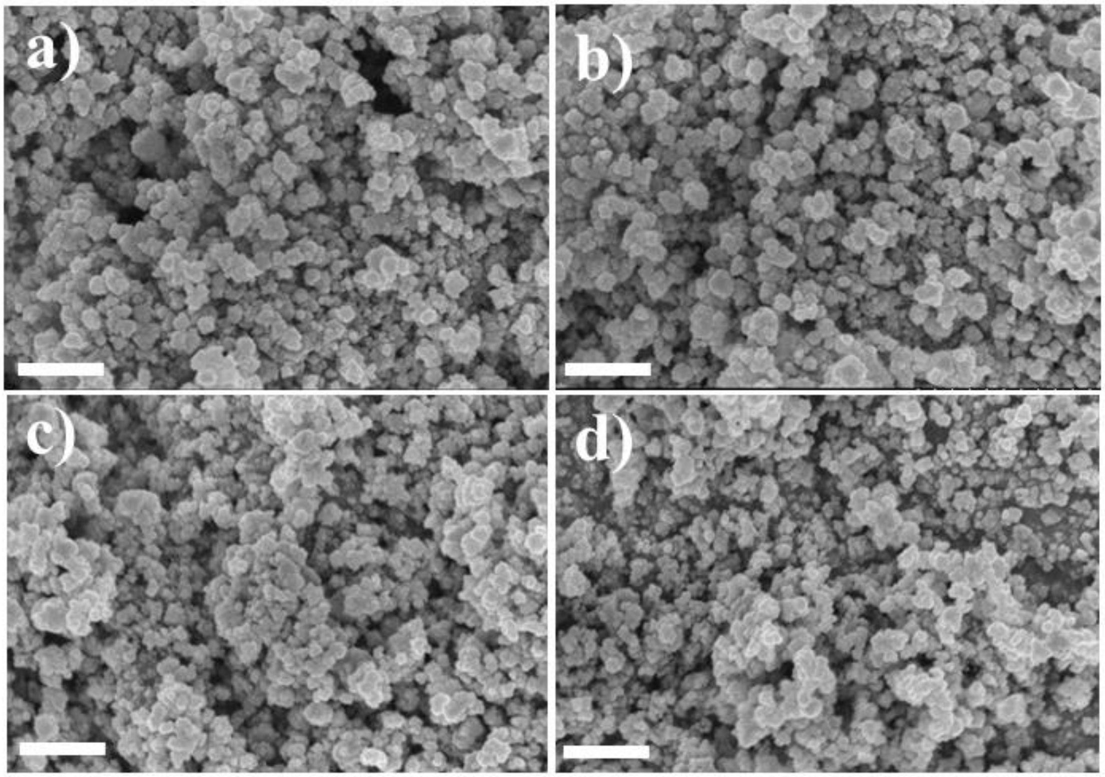

Supplementary Figure 4. SEM images of $Cr_{0.6}Ru_{0.4}O_2$ samples. (a) $Cr_{0.6}Ru_{0.4}O_2$ (450); (b) $Cr_{0.6}Ru_{0.4}O_2$ (500); (c) $Cr_{0.6}Ru_{0.4}O_2$ (600); (d) $Cr_{0.6}Ru_{0.4}O_2$ (650). Scale bars, 500 nm.

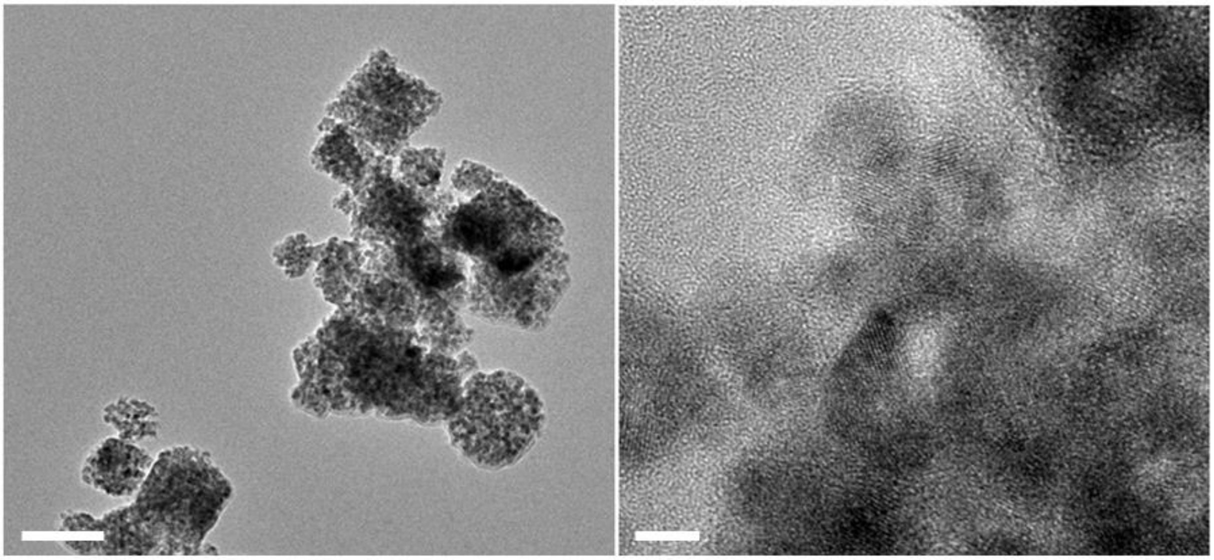

Supplementary Figure 5. TEM and HR-TEM images of $Cr_{0.6}Ru_{0.4}O_2$ (450). Scale bars, left (100 nm) and right (5 nm).

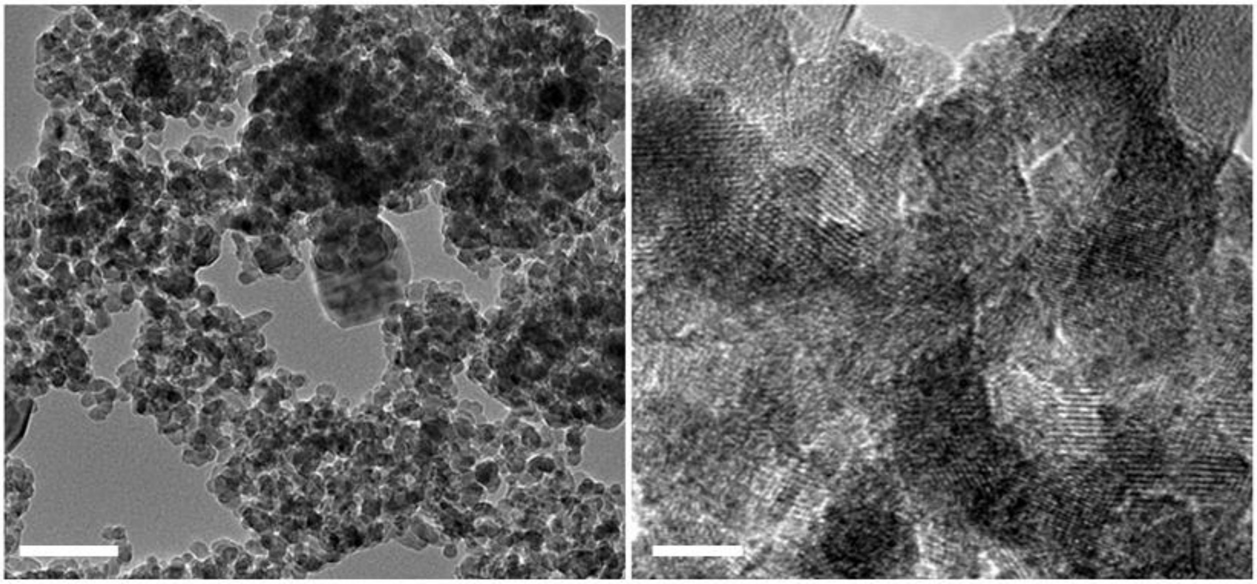

Supplementary Figure 6. TEM and HR-TEM images of $Cr_{0.6}Ru_{0.4}O_2$ (500). Scale bars, left (50 nm) and right (5 nm).

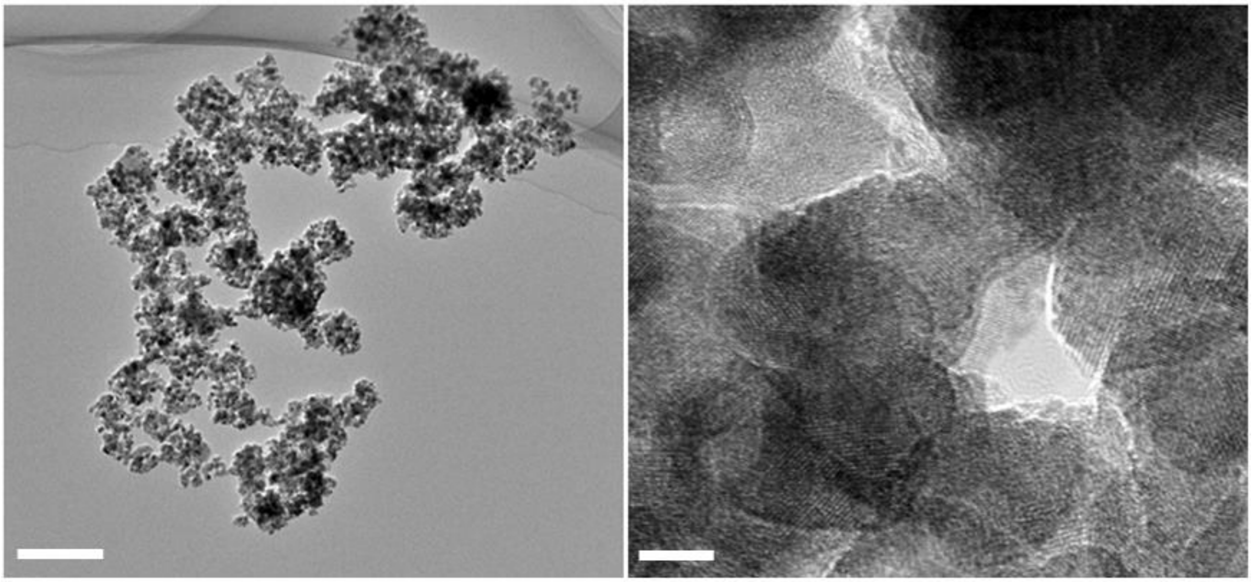

Supplementary Figure 7. TEM and HR-TEM images of $Cr_{0.6}Ru_{0.4}O_2$ (600). Scale bars, left (200 nm) and right (5 nm).

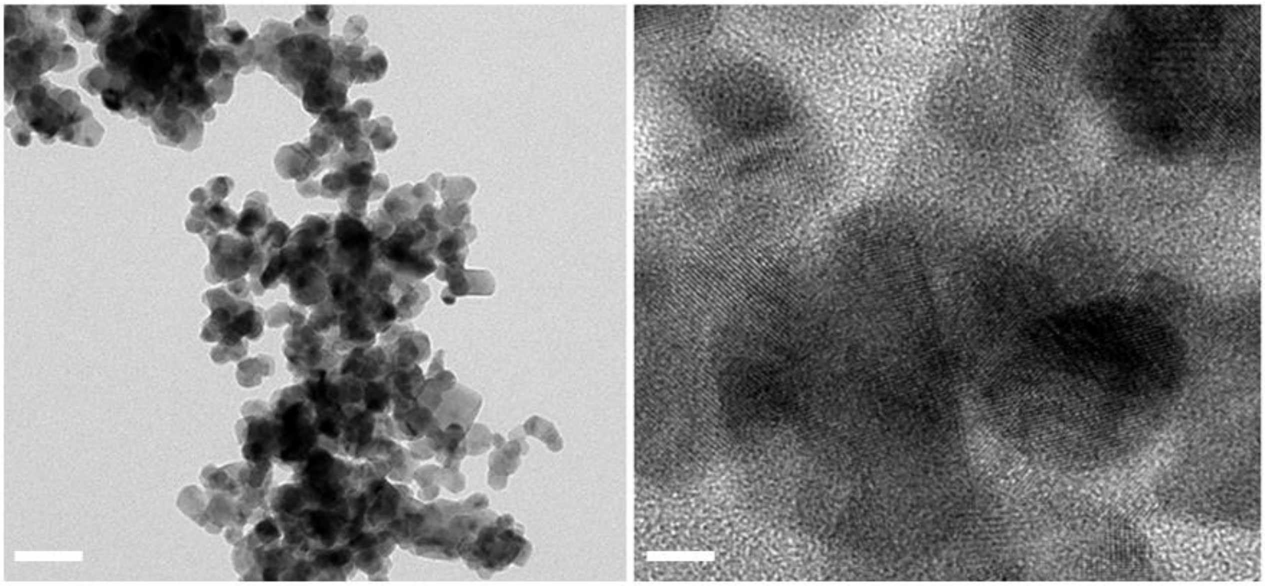

Supplementary Figure 8. TEM and HR-TEM images of $Cr_{0.6}Ru_{0.4}O_2$ (650). Scale bars, left (50 nm) and right (5 nm).

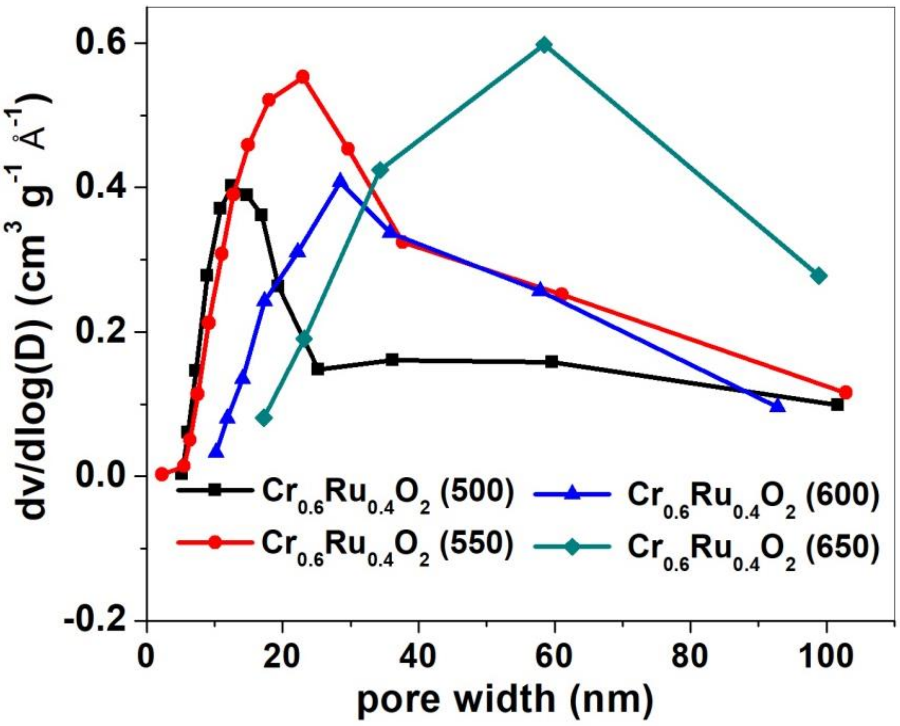

Supplementary Figure 9. BJH pore sizes distribution of $Cr_{0.6}Ru_{0.4}O_2$ powders.

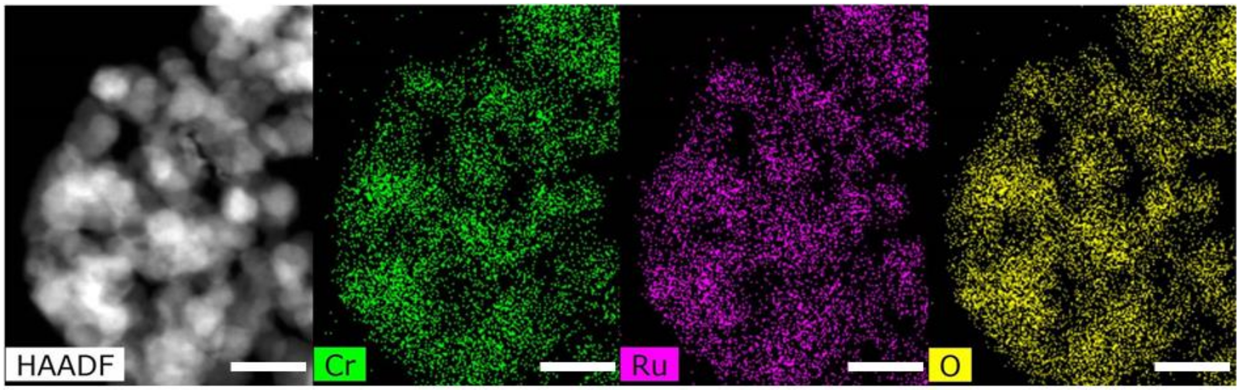

Supplementary Figure 10. HAADF-STEM image and EDS mapping of $Cr_{0.6}Ru_{0.4}O_2$(550). Scale bars, 20 nm.

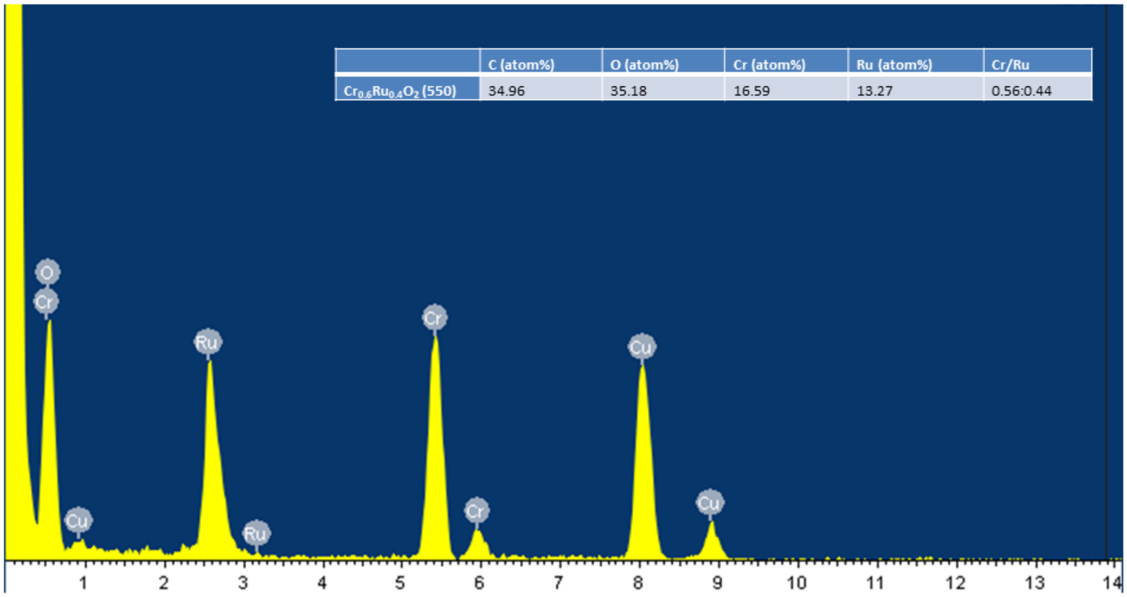

Supplementary Figure 11. EDS results of $Cr_{0.6}Ru_{0.4}O_2$(550), the element of Cu comes from carbon-coated copper grid.

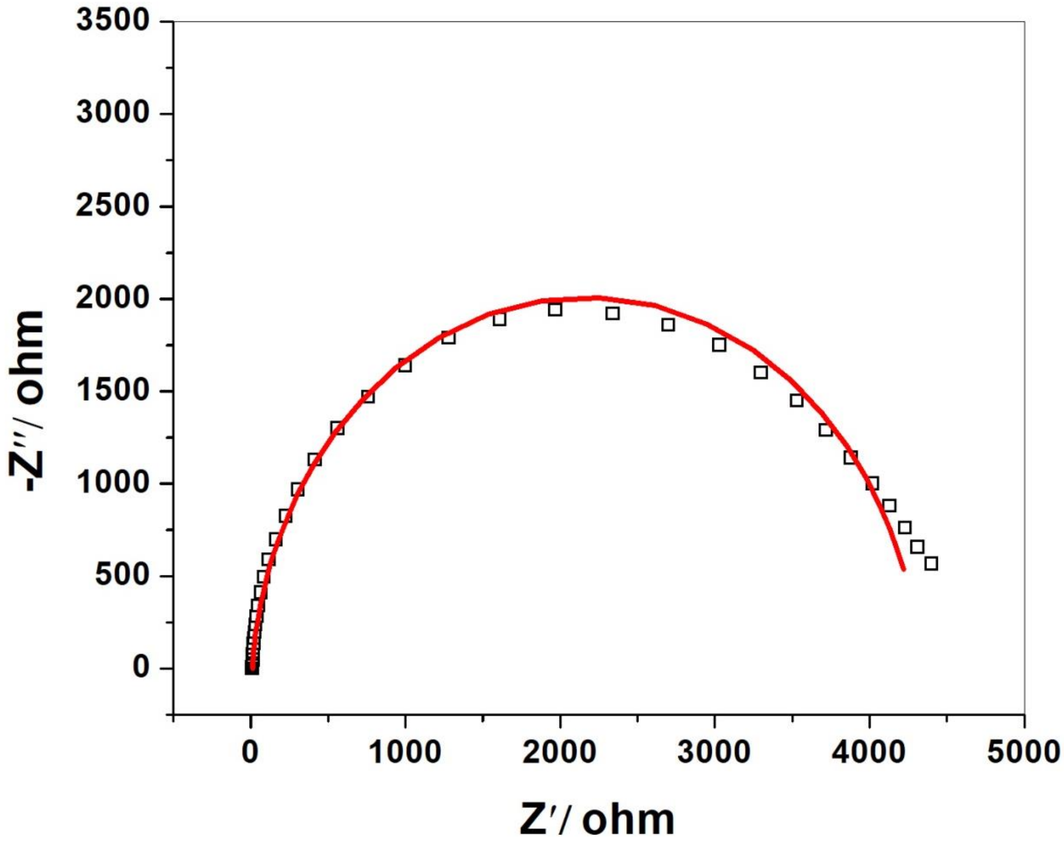

Supplementary Figure 12. Nyquist plots of RuO_2 at 1.395 V.

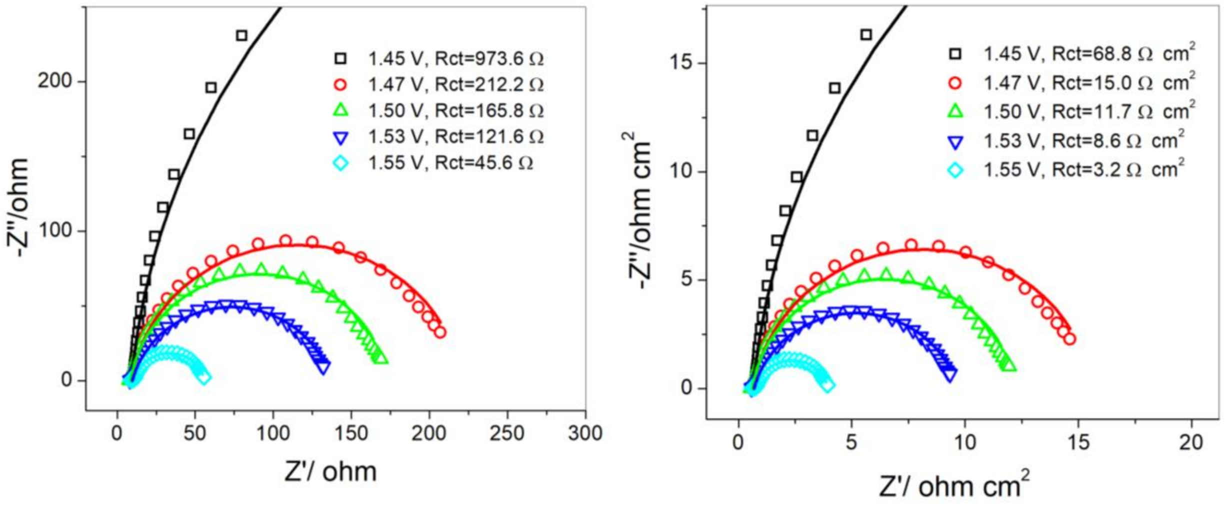

Supplementary Figure 13. Nyquist plots of RuO_2 at different voltage, (Left) Nyquist plots of RuO_2 at a series of voltages; (Right) The area-specific impedance (the electrode surface area is 0.07065 cm^2).

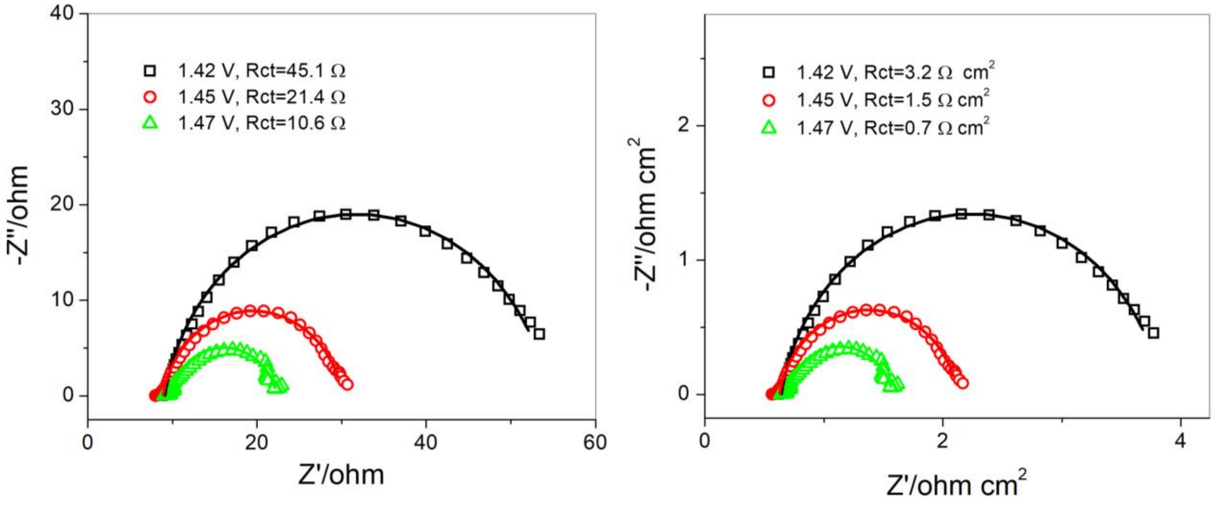

Supplementary Figure 14. Nyquist plots of $Cr_{0.6}Ru_{0.4}O_2$(550) at different voltage. (Left) Nyquist plots of $Cr_{0.6}Ru_{0.4}O_2$(550) at a series of voltages; (Right) The area-specific impedance (the electrode surface area is 0.07065 cm^2).

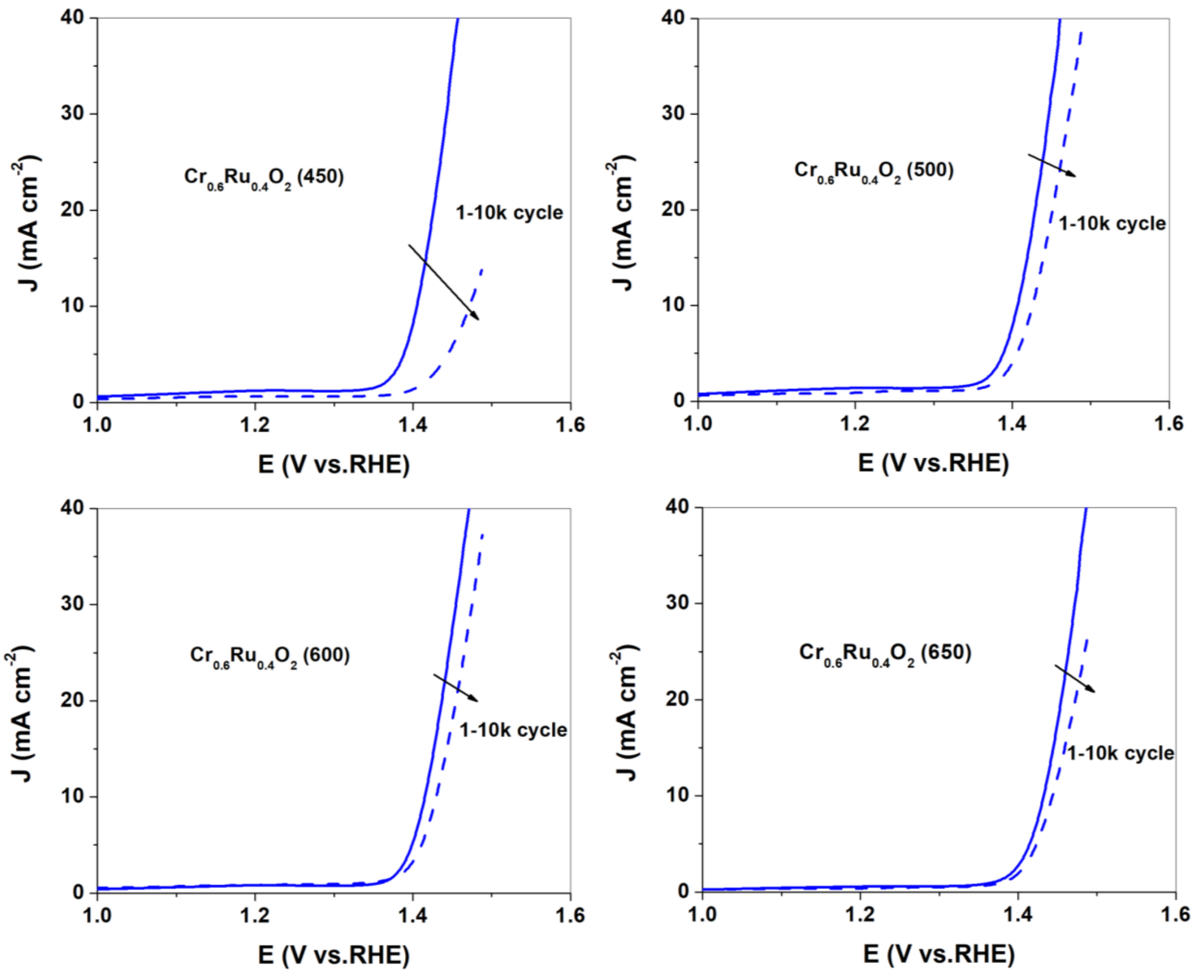

Supplementary Figure 15. LSVs of $Cr_{0.6}Ru_{0.4}O_2$ (450), $Cr_{0.6}Ru_{0.4}O_2$ (500), $Cr_{0.6}Ru_{0.4}O_2$ (600), and $Cr_{0.6}Ru_{0.4}O_2$ (650) electrocatalysts for the first cycle and 10,000th cycle.

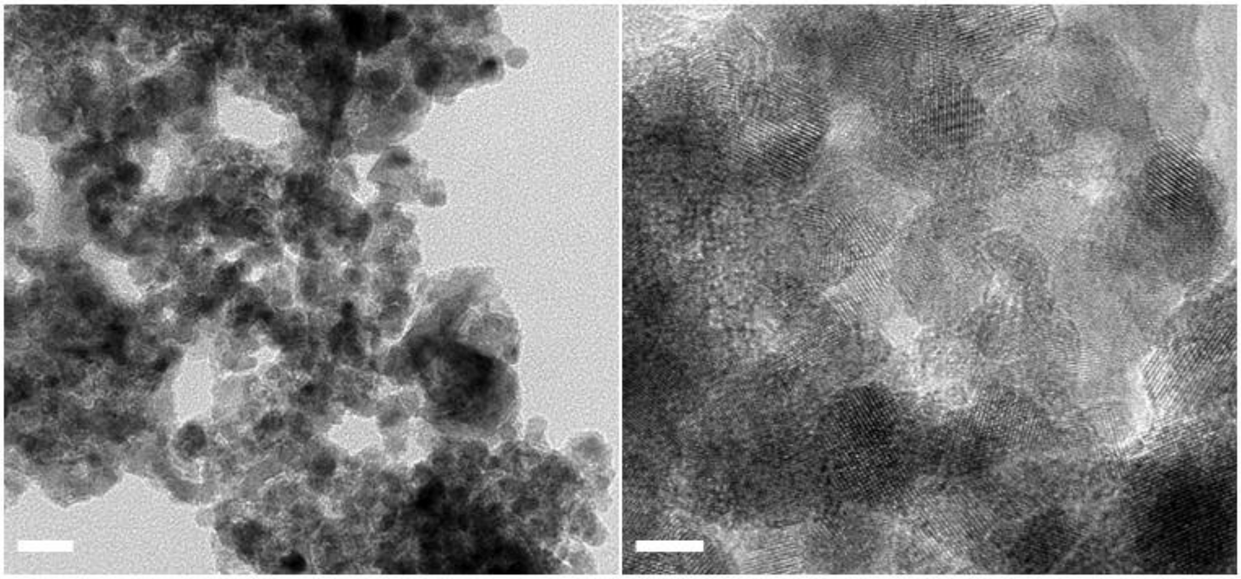

Supplementary Figure 16. TEM and HR-TEM images of $Cr_{0.6}Ru_{0.4}O_2$ (550) after 10,000 CV cycles. Scale bars, left (20 nm) and right (5 nm).

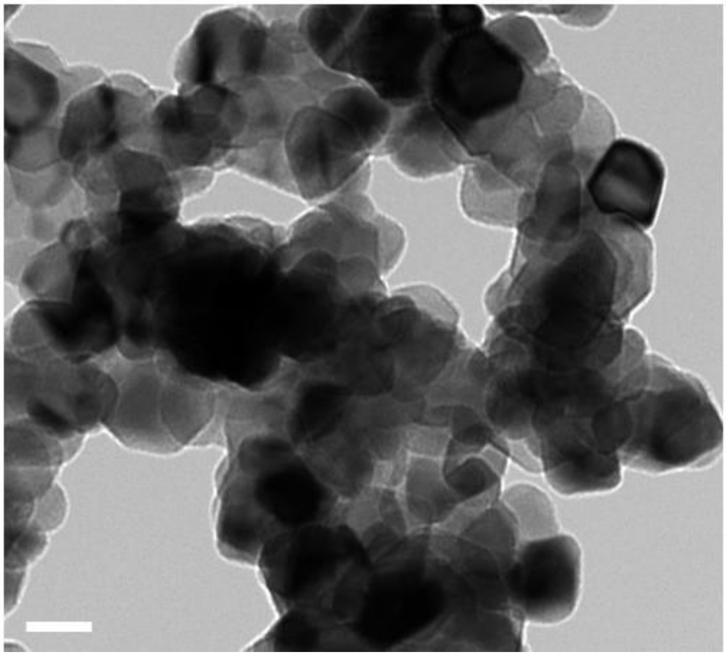

Supplementary Figure 17. TEM image of the commercial RuO_2 nanoparticles. Scale bar, 20 nm.

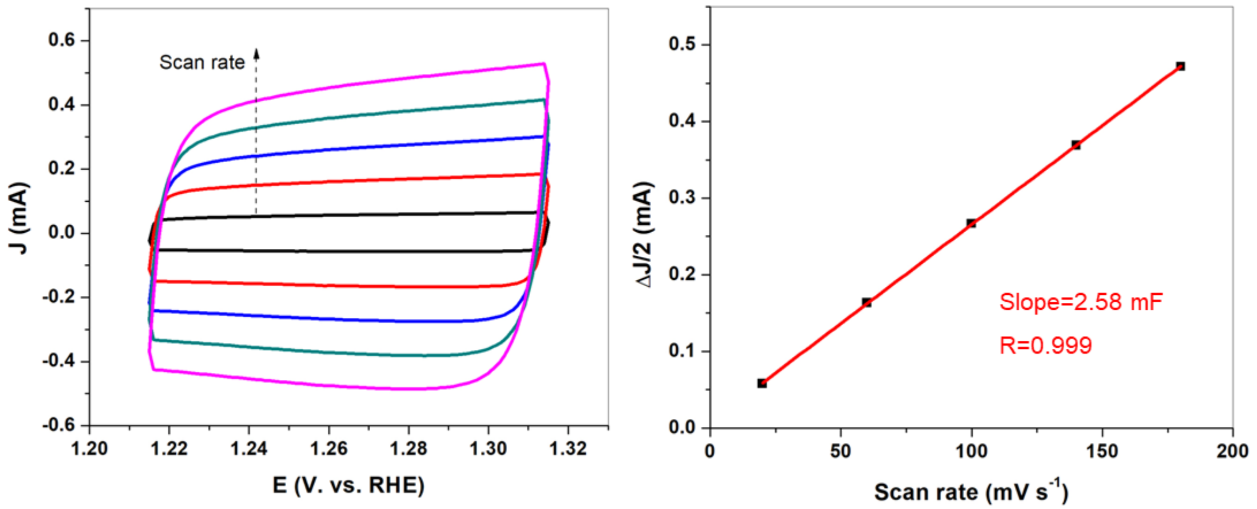

Supplementary Figure 18. Measurement of the double layer capacitance of $Cr_{0.6}Ru_{0.4}O_2$ (550). (Left) CVs of $Cr_{0.6}Ru_{0.4}O_2$ (550) collected at various scan rates (20, 60, 100, 140 and 180 mV s^{-1}); (Right) Capacitive current at 1.26 V (vs. RHE) against the scan rate and the corresponding C_{DL} value.

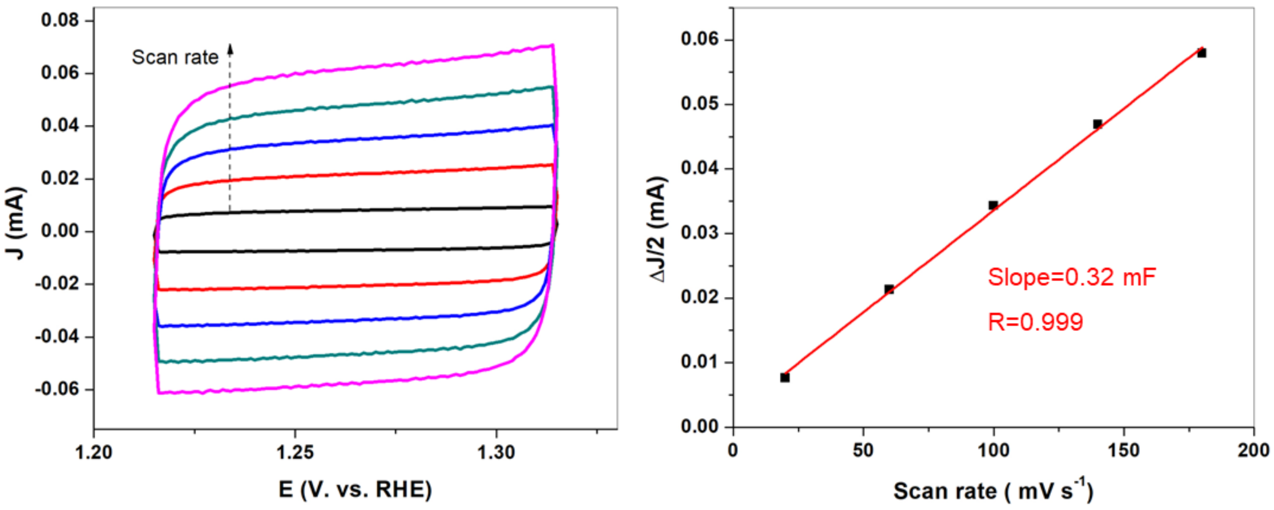

Supplementary Figure 19. Measurement of the double layer capacitance of RuO_2. (Left) CVs of RuO_2 collected at various scan rates (20, 60, 100, 140 and 180 mV s^{-1}); (Right) Capacitive current at 1.26 V (vs. RHE) against the scan rates and the corresponding C_{DL} value.

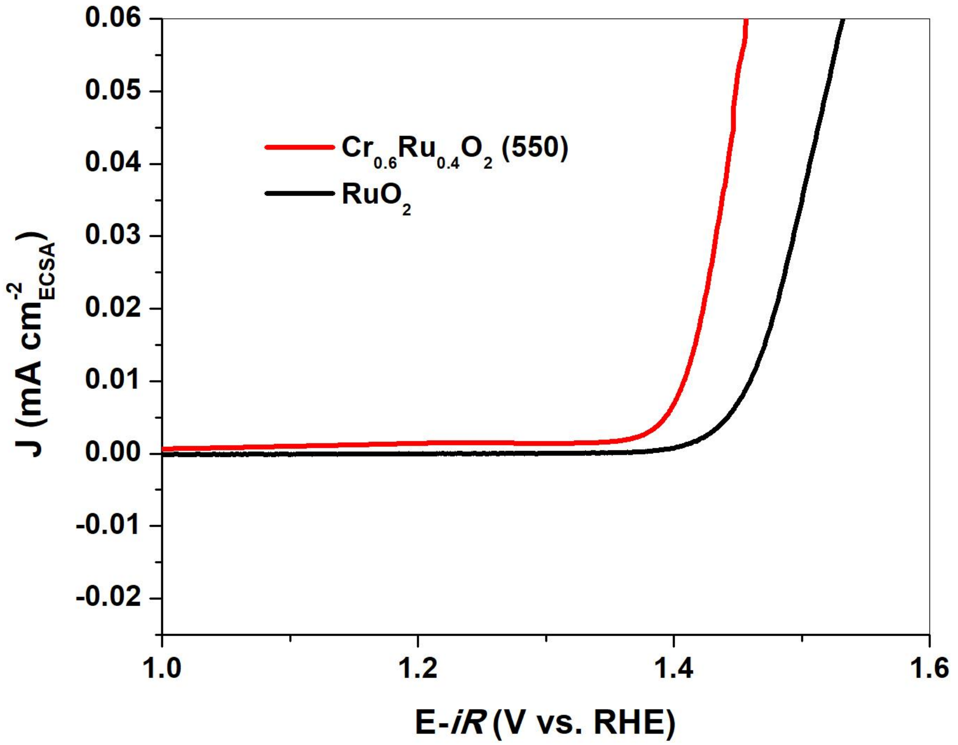

Supplementary Figure 20. ECSA based LSVs of $Cr_{0.6}Ru_{0.4}O_2$(550) and RuO_2.

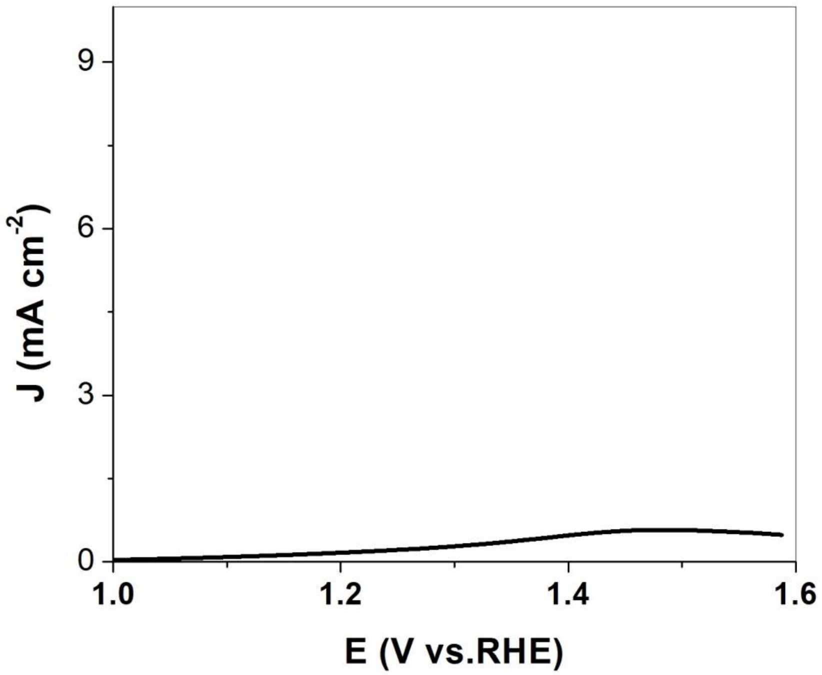

Supplementary Figure 21. LSV of commerical CrO_2 at the first run.

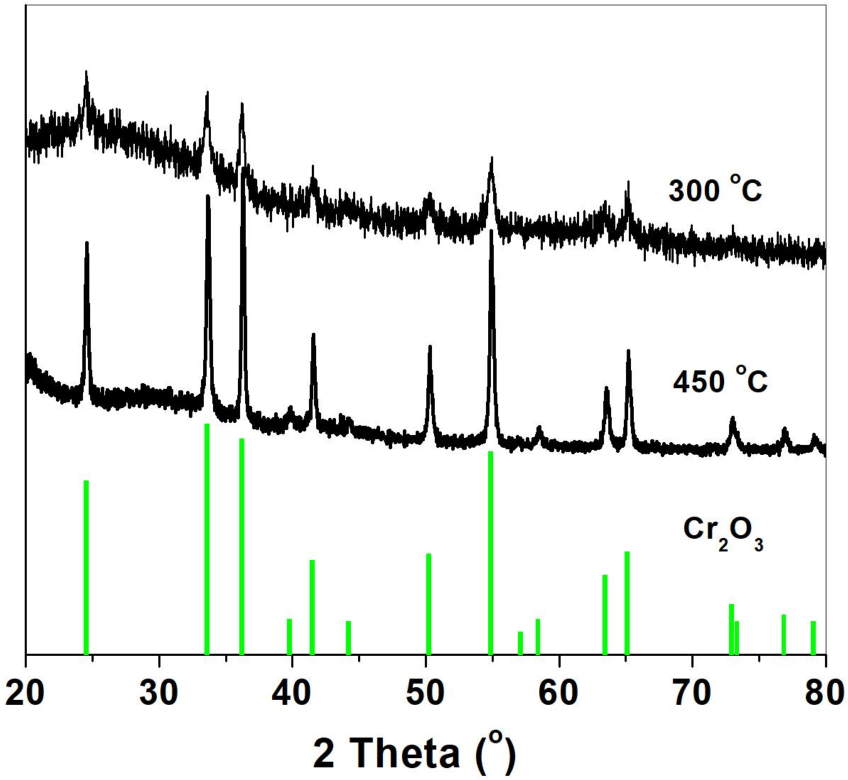

Supplementary Figure 22. The PXRD pattern of the product by annealing pure MIL-101 (Cr) without loading $RuCl_3$ at 300 and 450 ^oC. The reference PXRD pattern of Cr_2O_3 is obtained from Jade 2004 (JCPDS No. 06-0504).

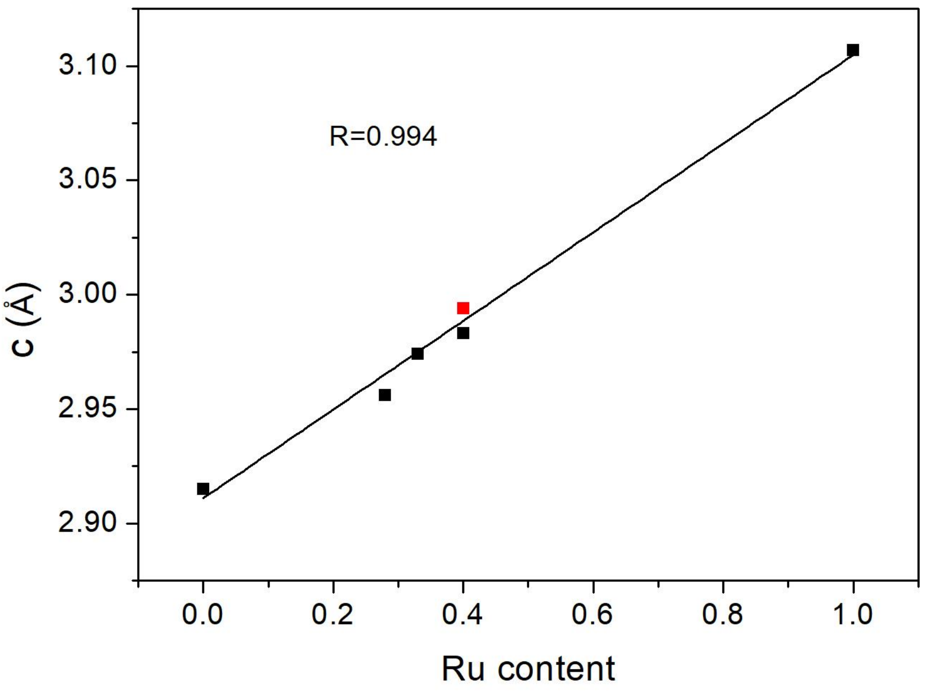

Supplementary Figure 23. Relationship between the lattice parameter c and Ru content. The red point was the data of $Cr_{0.6}Ru_{0.4}O_2$ obtained by Retiveld refinement based on a high quality PXRD with a very slow scan. For consistent with other samples, this point wasn't used for the linear fitting.

Data description of Supplementary Figure 23:

The calculated lattice parameters of $Cr_{1-x}Ru_xO_2$ with solid solution as the major or pure phase:

$Cr_{0.72}Ru_{0.28}O_{2-\delta}$, a=b=4.483 Å, c=2.955 Å

$Cr_{0.67}Ru_{0.33}O_2$, a=b=4.495 Å, c=2.974 Å

$Cr_{0.6}Ru_{0.4}O_2$, a=b=4.497 Å, c=2.983 Å. For this sample, as we have presented in our manuscript, the lattice parameters by Retiveld refinement based on a high quality PXRD with a very slow scan were: a=b=4.495 Å, c=2.994 Å. We also added this point in the following plot (lattice parameter c vs. Ru content), but for consistent with other samples, we don't use this data for the linear fitting.

The standard parameters of RuO_2 and CrO_2:

RuO_2, a=b=4.499 Å, c=3.107 Å

CrO_2, a=b=4.421 Å, c=2.916 Å

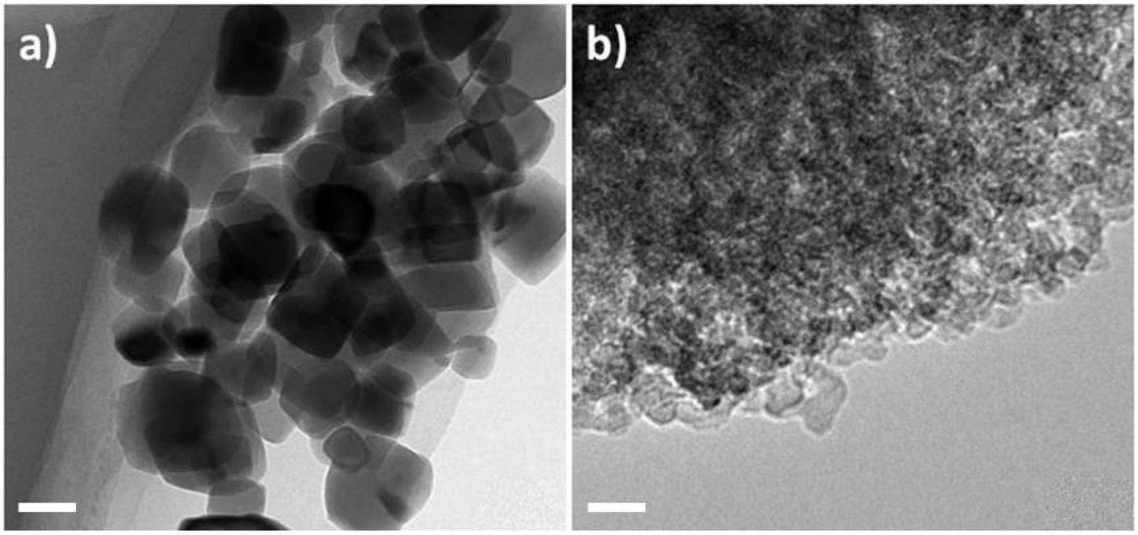

Supplementary Figure 24. TEM images of Cr_2O_3 by directly annealing MIL-101(Cr) at different temperatures. (a) 450 ^{o}C; (b) 300 ^{o}C. Scale bars, left (50 nm) and right (5 nm).

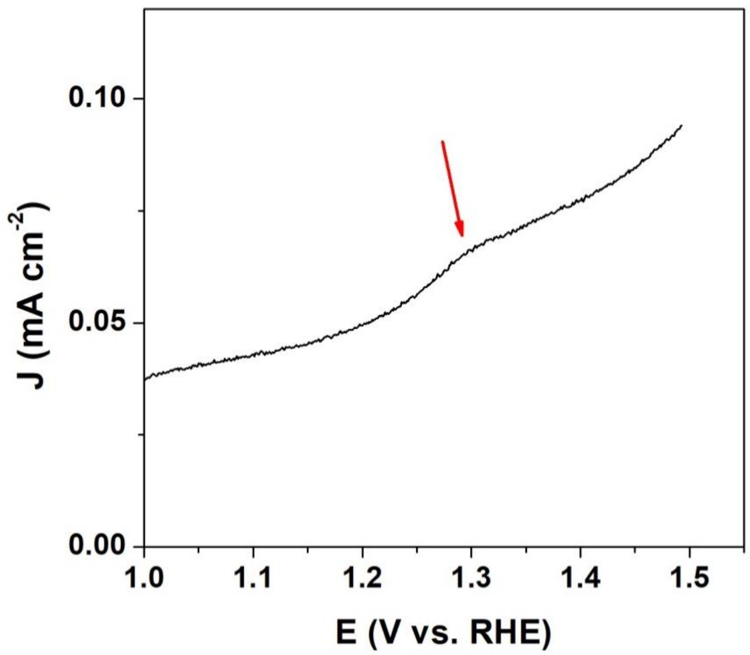

Supplementary Figure 25. LSV curve of Cr_2O_3 obtained from annealing MIL-101(Cr) at 300 ^{o}C.

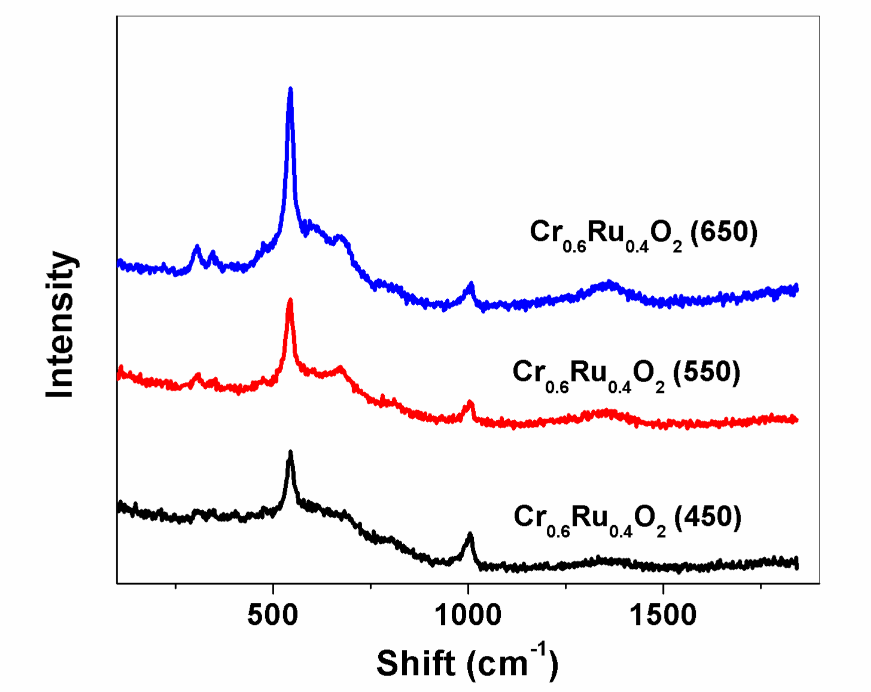

Supplementary Figure 26. Raman spectroscopy of $Cr_{0.6}Ru_{0.4}O_2$ (450), $Cr_{0.6}Ru_{0.4}O_2$ (550) and $Cr_{0.6}Ru_{0.4}O_2$ (650).

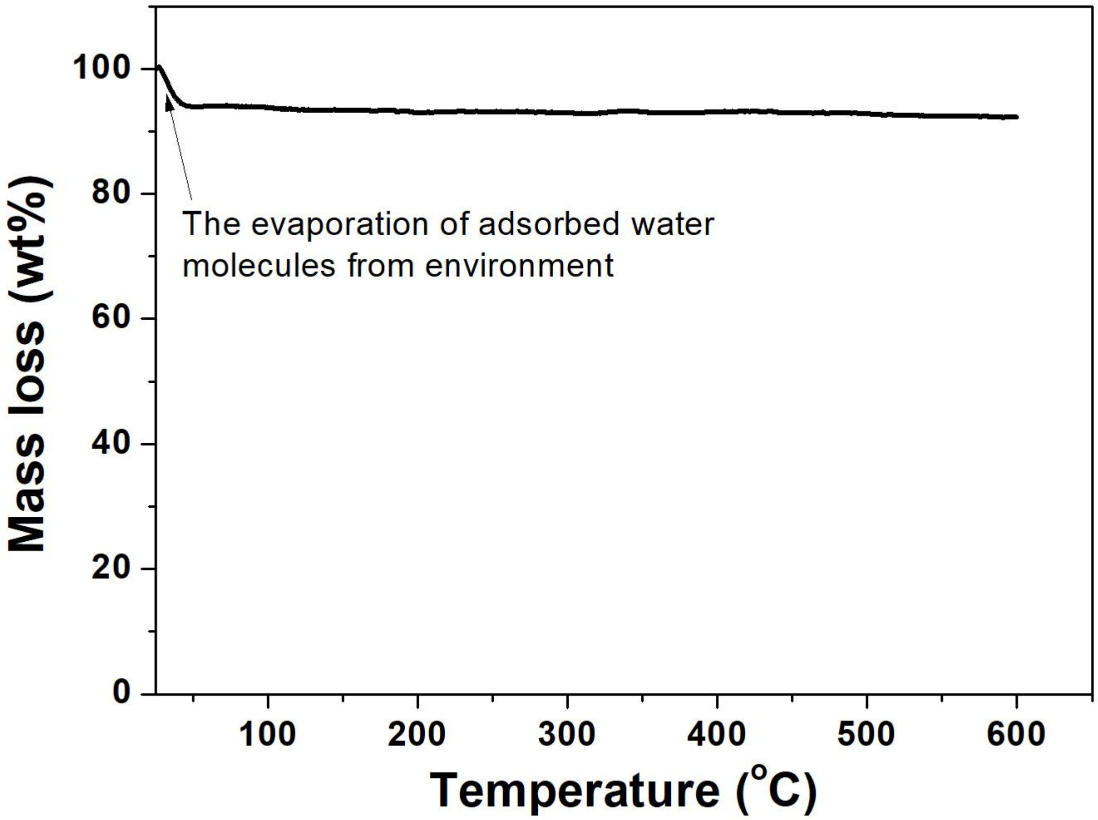

Supplementary Figure 27. TG profile of $Cr_{0.6}Ru_{0.4}O_2$ (450) in air with a heating rate of 5 ^{o}C/min.

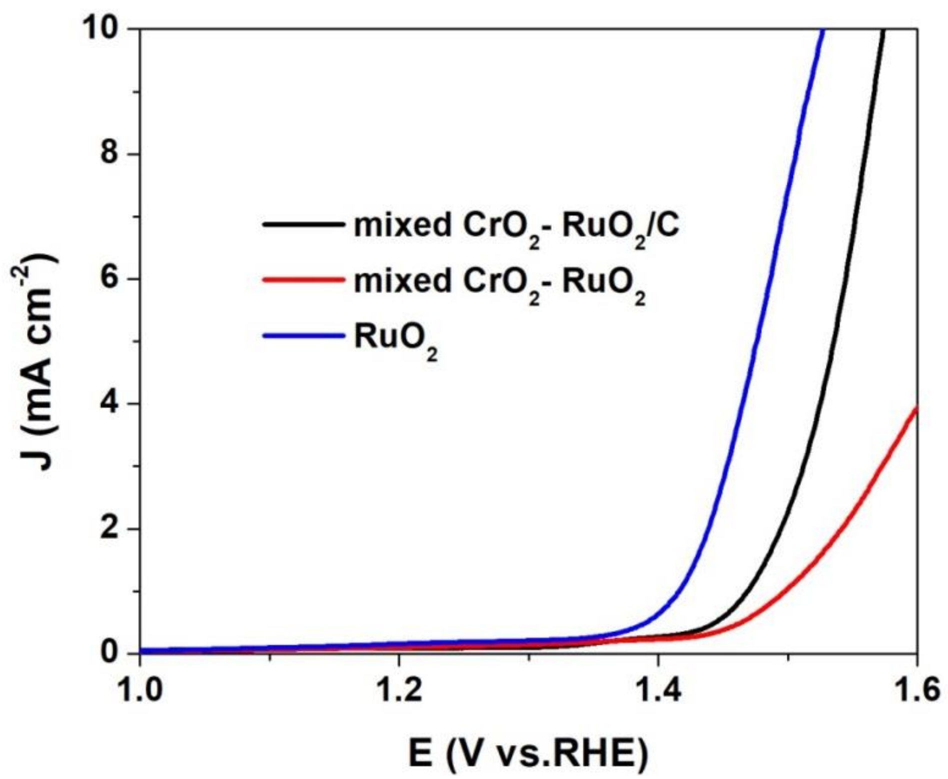

Supplementary Figure 28. LSV curves of pure RuO_2, mixed CrO_2-RuO_2 and CrO_2-RuO_2/C.

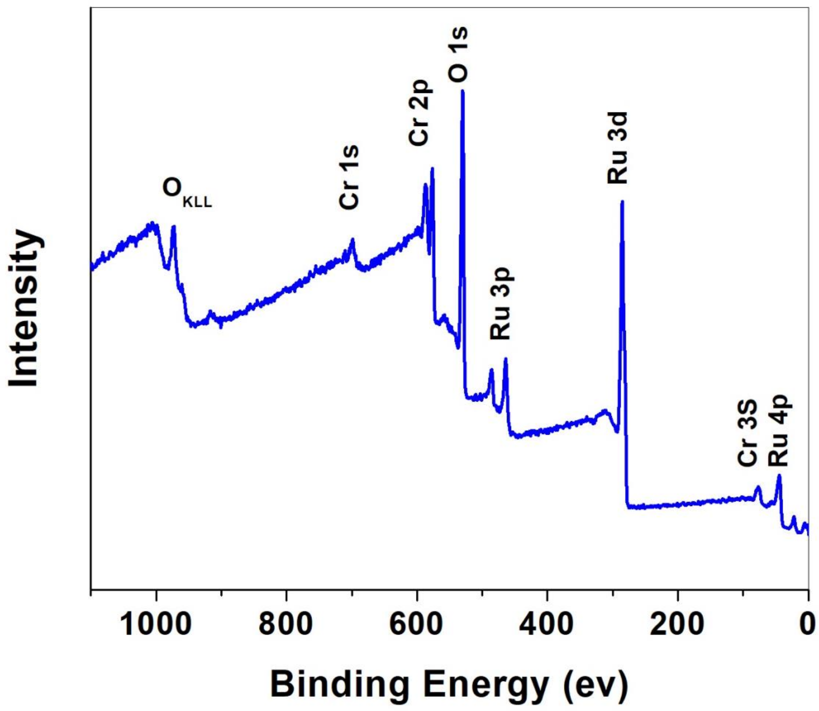

Supplementary Figure 29. XPS wide-scan spectra of $Cr_{0.6}Ru_{0.4}O_2$ (550).

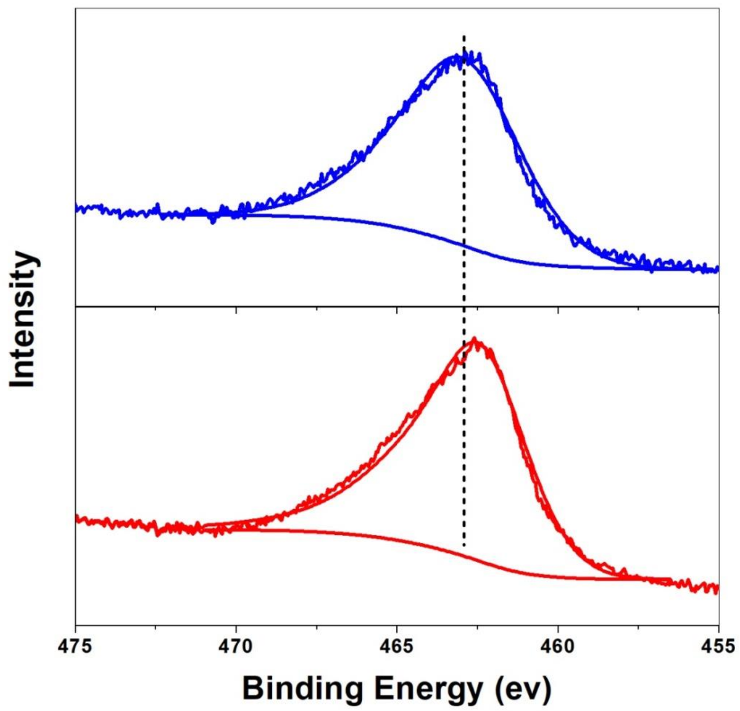

Supplementary Figure 30. XPS spectra of $Cr_{0.6}Ru_{0.4}O_2$ (550) (top) and RuO_2 (bottom) for Ru 3p.

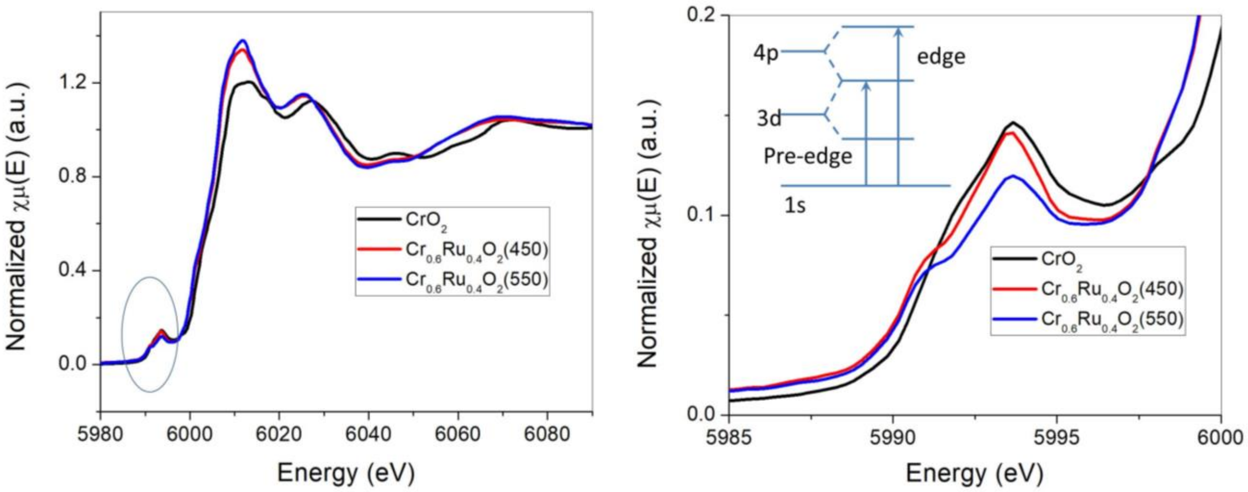

Supplementary Figure 31. XAS analysis of $Cr_{0.6}Ru_{0.4}O_2$ (450) and $Cr_{0.6}Ru_{0.4}O_2$ (550). (left) Normalized Cr K-edge XANES spectra for $Cr_{0.6}Ru_{0.4}O_2$ (450), $Cr_{0.6}Ru_{0.4}O_2$ (550) and reference CrO_2. (Right) Shows the amplified pre-edge region and the inset diagram shows the energy level of possible transitions.

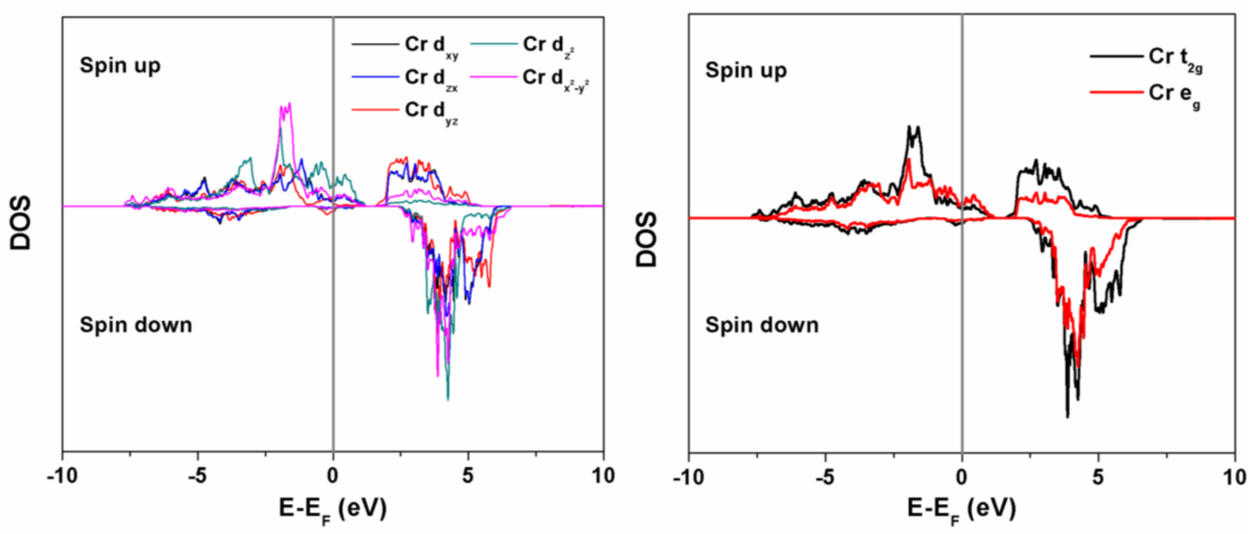

Supplementary Figure 32. Projected density of states (PDOS) of Cr in $Cr_5Ru_3O_{16}$. (Left) PDOS of Cr atom of $Cr_5Ru_3O_{16}$; (Right) PDOS of Cr t_{2g} and e_g of $Cr_5Ru_3O_{16}$.

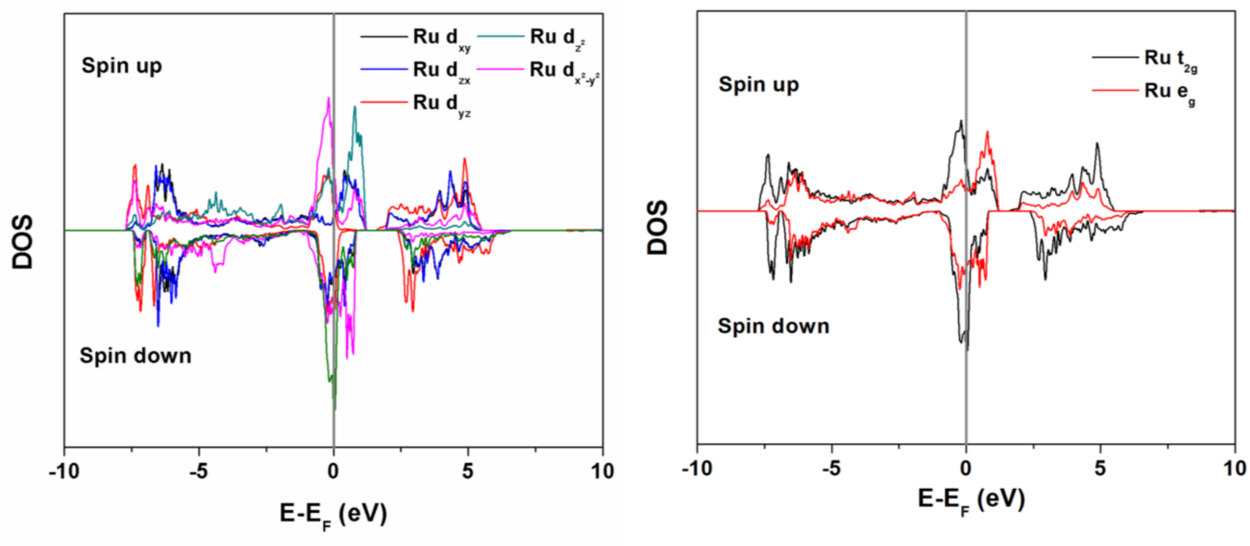

Supplementary Figure 33. PDOS of Ru in $Cr_5Ru_3O_{16}$. (Left) PDOS of Ru atom of $Cr_5Ru_3O_{16}$; (Right) PDOS of Ru t_{2g} and e_g of $Cr_5Ru_3O_{16}$.

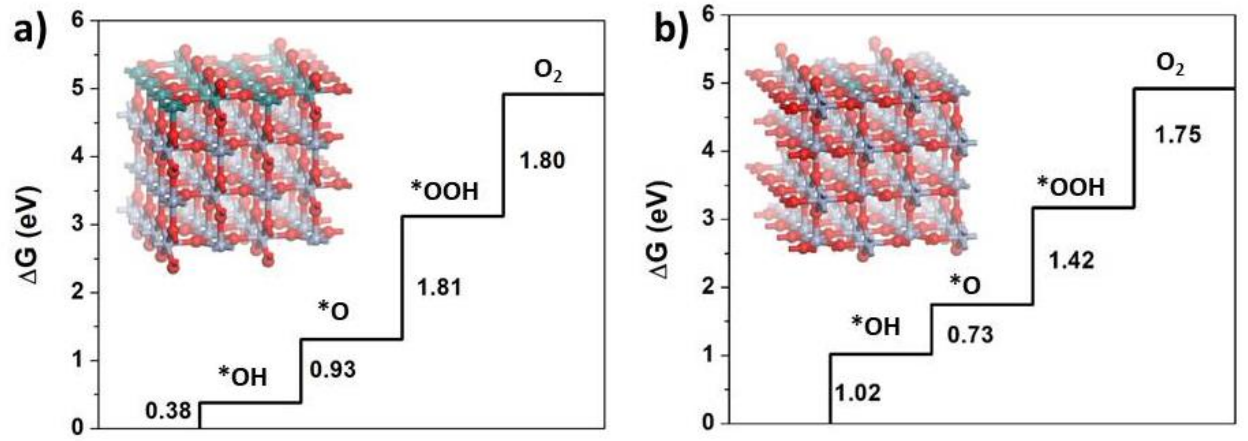

Supplementary Figure 34. Energy profiles of OER processes on CrO_2(110) surfaces. (a) Coated RuO_2 layer; (b) Doped Ru atom in surface. The simulated models are depicted in insets. Color code: red, O; cyan, Ru; gray, Cr.

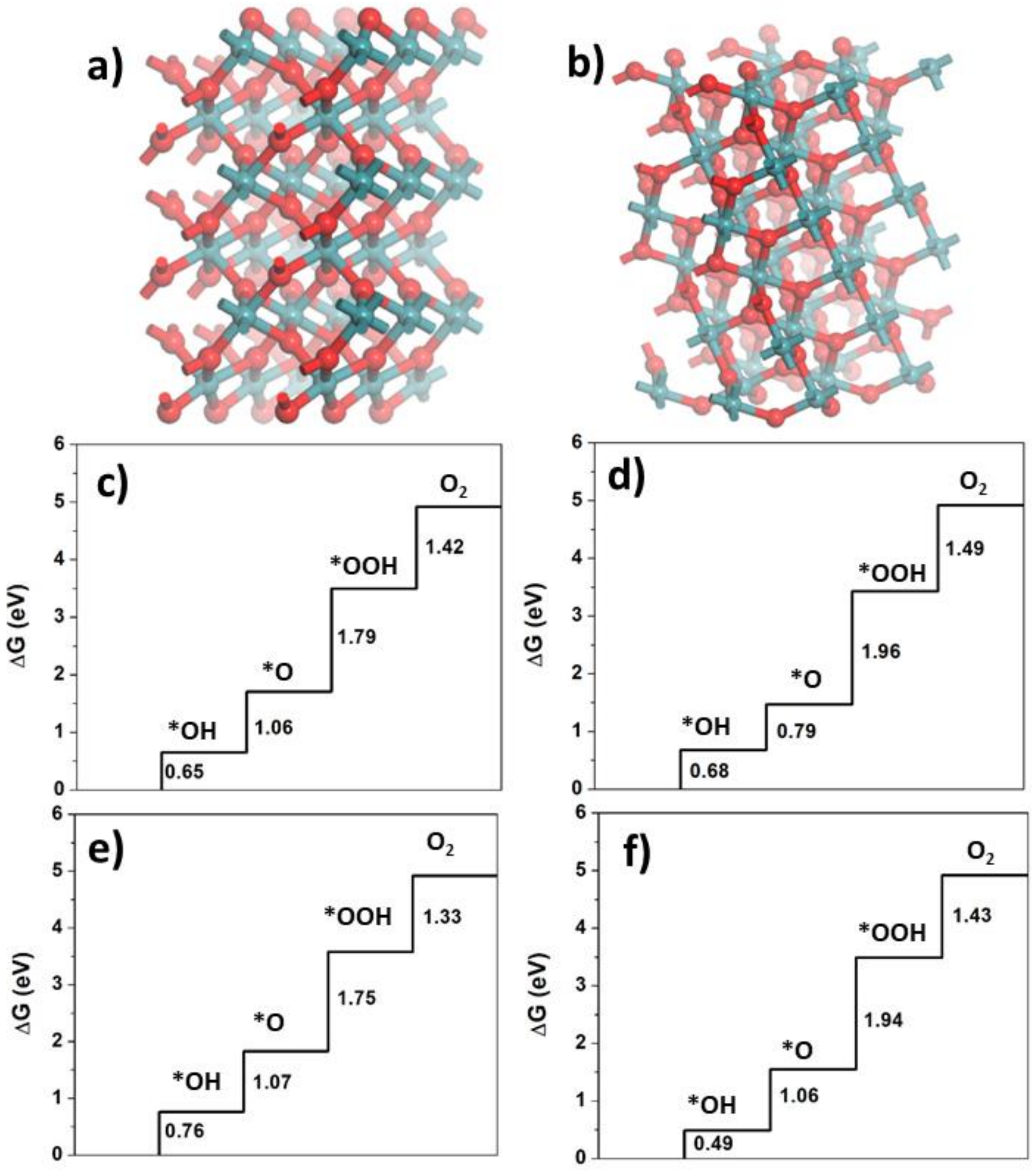

Supplementary Figure 35. Energy profiles of OER processes on various slab models. (a-b) (200) and (101) facets of rutile crystal; (c) RuO_2(200); (d) RuO_2(101); (e) RuO_2-CrO_2(200); (f) RuO_2-CrO_2 (101).

Supplementary Table 1. Lattice parameters of $Cr_{0.6}Ru_{0.4}O_2$.

Formula	$Cr_{0.6}Ru_{0.4}O_2$
Crystal system	tetragonal
Space group	P 42/m n m
a/Å	4.49518
b/Å	4.49518
c/Å	2.99446
α	90°
β	90°
γ	90°
Cell volume/ $Å^3$	60.50791

Supplementary Table 2. BET surface areas of $Cr_{0.6}Ru_{0.4}O_2$ powders and RuO_2.

	$Cr_{0.6}Ru_{0.4}O_2$ (500)	$Cr_{0.6}Ru_{0.4}O_2$ (550)	$Cr_{0.6}Ru_{0.4}O_2$ (600)	$Cr_{0.6}Ru_{0.4}O_2$ (650)	Commercial RuO_2
BET surface area	72.5 m^2 g^{-1}	86.1 m^2 g^{-1}	50.1 m^2 g^{-1}	50.8 m^2 g^{-1}	8.9 m^2 g^{-1}

Supplementary Table 3. Parameters obtained by fitting EIS experimental spectra recorded at 1.395 V.

Catalysts	R_{sol} (ohm)	R_{ct} (ohm)	CPE-T	CPE-P
$Cr_{0.6}Ru_{0.4}O_2$ (450)	9.4	69.5	6.4E-03	9.0E-01
$Cr_{0.6}Ru_{0.4}O_2$ (500)	10.8	80.1	13.1E-03	8.2E-01
$Cr_{0.6}Ru_{0.4}O_2$ (550)	7.9	97.2	13.7E-03	8.8E-01
$Cr_{0.6}Ru_{0.4}O_2$ (600)	9.3	175.3	8.6E-03	8.7E-01
$Cr_{0.6}Ru_{0.4}O_2$ (650)	9.4	278.1	6.7E-03	8.8E-01
RuO_2	9.6	4317	4.2E-04	9.5E-01

Supplementary Table 4. ICP-MS analysis of dissolved Ru and Cr ions after 10,000 CV cycles in 0.5 M H_2SO_4.

Sample amount	20 ug	20 ug	40 ug	60 ug
Concentration of Ru ion (ppb)	1.92	2.15	2.6	5.04
Concentration of Cr ion (ppb)	4.33	5.08	9.02	16.12
Loss of mass (Ru)	2.47%	2.76%	1.66%	2.16%
Loss of mass (Cr)	7.19%	8.42%	7.44%	7.02%
Average mass loss (Ru)	2.26%			
Average mass loss (Cr)	7.51%			

Supplementary Table 5. The OER activity of RuO_2 reported in literatures.

Catalyst	substrate	Electrolyte	Overpotential at specific current	Stability	Reference
RuO_2	GCE	0.5 M H_2SO_4	297@10 mA cm^{-2} 240@1 mA cm^{-2}	Chronopotentiometry @10 mA cm^{-2}: full loss activity in 1 hour	This work
RuO_2	GCE	0.1 M $HClO_4$	320 @ 1 mA cm^{-2}	Chronopotentiometry @ 1 mA cm^{-2}: full loss activity in 1.5 hours	*J. Am. Chem. Soc. 2017*, *139*, *12076.*
RuO_2	GCE	0.1 M $HClO_4$	430@10 mA cm^{-2}	--	*J. Phys. Chem. Lett. 2012, 3, 399.*
RuO_2	Ti	0.5 M H_2SO_4	240@1 mA cm^{-2}	--	*Electrochimica Acta. 1998, 44, 1515.*
RuO_2	Au	0.5 M H_2SO_4	230@1 mA cm^{-2}	CV measurements: activity dramatically decreased in 1000 cycles	*J. Phys. Chem. C, 2016, 120, 2562–2573.*
RuO_2	GCE	0.5 M H_2SO_4	289@10 mA cm^{-2}	CV measurements: activity dramatically decreased in 2000 cycles	*J. Mater. Chem. A 2017, 5, 17221.*

Supplementary Table 6. ECSA parameters for $Cr_{0.6}Ru_{0.4}O_2$(550) and RuO_2.

Catalyst	C_{DL}	ECSA	RF
$Cr_{0.6}Ru_{0.4}O_2$(550)	2.58 mF	73.7 cm^2	1043
RuO_2	0.32 mF	9.1 cm^2	129

Supplementary Table 7. The comparison of overpotentials of representative OER electrocatalysts in acidic media.

Catalyst	substrate	Electrolyte	Overpotential at 10 mA cm^{-2} (mV)	Chronopotentiometry at specific current density	Mass activity at 270 mV	Ref.
$Cr_{0.6}Ru_{0.4}O_2$(550)	GCE	0.5 M H_2SO_4	178	10 h @10 mA cm^{-2}	229 A g^{-1}	This work
Ba[Co-POM]	CP	1 M H_2SO_4	361	--	--	1
IrCoNi PHNCs	GCE	0.1 M $HClO_4$	303	3.3 h@5 mA cm^{-2}	39.2 A g^{-1}	2
IrNiCu DNF	GCE	0.1 M $HClO_4$	300	--	124.8 A g^{-1}_{Ir}	3
Ir	GF	0.5 M H_2SO_4	290	10 h@10 mA cm^{-2}	--	4
$Ni_{0.5}Mn_{0.5}Sb_{1.7}Oy$	ATO	1 M H_2SO_4	~672	170 h@10 mA cm^{-2}	--	5
$W_{0.57}Ir_{0.43}O_{3-\delta}$	FTO	1 M H_2SO_4	370	0.6 h@10 mA cm^{-2}	--	6
Co_3O_4	FTO	0.5 M H_2SO_4	570	12 h@10 mA cm^{-2}	--	7
$Y_2Ru_2O_{7-\delta}$	GCE	0.1 M $HClO_4$	270@1 mA cm^{-2}	8 h@1 mA cm^{-2}	19.6 A g^{-1}	8
IrO_2-RuO_2@Ru	GCE	0.5 M H_2SO_4	281	--	13.2 A g^{-1}	9
NiFeP	Free-standing	0.05 M H_2SO_4	540	12 h@10 mA cm^{-2}	--	10
IrNi NCs	GCE	0.1 M $HClO_4$	280	2 h@5 mA cm^{-2}	--	11
Co-IrCu ONC	GCE	0.1 M $HClO_4$	290	--	170 A g^{-1}_{Ir}	12
IrO_x-Ir	GC plates	0.5 M H_2SO_4	290	100 h@2 mA cm^{-2}	28.2 A g^{-1}	13
$IrO_x/SrIrO_3$	$SrTiO_3$	0.5 M H_2SO_4	270-290	30 h@10 mA cm^{-2}	--	14
$BaYIrO_6$	Au	0.1 M H_4ClO_4	~315	1 h@10 mA cm^{-2}	10 A g^{-1}	15
Ir-Ni Oxide	Ti	0.1 M HClO4	~270	--	--	16
$IrNiO_x$	ATO	0.05 M H_2SO_4	~330	--	98 A g^{-1}_{Ir}	17
IrO_2	GCE	0.1 M H_4ClO_4	~430	--	--	18

Supplementary Table 8. Surface energy of a series of facets of RuO_2.

Facet	Surface energy / eV
**(110)**	**1.41**
**(101)**	**1.50**
**(200)**	**1.67**
(001)	1.79
(111)	1.99
(210)	2.34
(211)	2.48

Supplementary Table 9. Screening of rutile-like RuO_2-MO_2 systems based on DFT calculations.

	n(valence *e*)	electronegativity	a / Å	c / Å	Partial charge on Ru / |*e*|
RuO_2	-	1.42	4.497	3.115	1.73
M=					
Ti	4	1.32	4.643	2.961	1.79
V	5	1.45	4.521	3.053	1.77
**Cr**	**6**	**1.56**	**4.535**	**3.009**	**1.92**
Mn	7	1.60	4.495	3.087	1.88
Ge	4	2.02	4.496	3.026	1.71
Nb	5	1.23	4.680	3.118	1.60
Mo	6	1.30	4.606	3.152	1.71
Rh	9	1.45	4.501	3.109	1.82
Sn	4	1.72	4.650	3.211	1.78
W	6	1.40	4.592	3.198	1.52
Pb	6	1.55	4.702	3.245	1.76

Supplementary References

1. Blasco-Ahicart, M., Soriano-Lopez, J., Carbo, J. J., Poblet, J. M. & Galan-Mascaros, J. R. Polyoxometalate electrocatalysts based on earth-abundant metals for efficient water oxidation in acidic media. *Nat. Chem.* **10**, 24-30 (2018).
2. Feng, J., Lv, F., Zhang, W., Li, P., Wang, K., Yang, C., Wang, B., Yang, Y., Zhou, J., Lin, F., Wang, G. C. & Guo, S. Iridium-based multimetallic porous hollow nanocrystals for efficient overall-water-splitting catalysis. *Adv. Mater.* **29**, 1703798 (2017).
3. Park, J., Sa, Y. J., Baik, H., Kwon, T., Joo, S. H. & Lee, K. Iridium-based multimetallic nanoframe@nanoframe structure: An efficient and robust electrocatalyst toward oxygen evolution reaction. *ACS Nano* **11**, 5500-5509 (2017).
4. Zhang, J., Wang, G., Liao, Z., Zhang, P., Wang, F., Zhuang, X., Zschech, E. & Feng, X. Iridium nanoparticles anchored on 3D graphite foam as a bifunctional electrocatalyst for excellent overall water splitting in acidic solution. *Nano Energy* **40**, 27-33 (2017).
5. Moreno-Hernandez, I. A., MacFarland, C. A., Read, C. G., Papadantonakis, K. M., Brunschwig, B. S. & Lewis, N. S. Crystalline nickel manganese antimonate as a stable water-oxidation catalyst in aqueous 1.0 M H_2SO_4. *Energy Environ. Sci.* **10**, 2103-2108 (2017).
6. Kumari, S., Ajayi, B. P., Kumar, B., Jasinski, J. B., Sunkara, M. K. & Spurgeon, J. M. A low-noble-metal $W_{1-x}Ir_xO_{3-\delta}$ water oxidation electrocatalyst for acidic media *via* rapid plasma synthesis. *Energy Environ. Sci.* **10**, 2432-2440 (2017).
7. Mondschein, J. S., Callejas, J. F., Read, C. G., Chen, J. Y. C., Holder, C. F., Badding, C. K. & Schaak, R. E. Crystalline cobalt oxide films for sustained electrocatalytic oxygen evolution under strongly acidic conditions. *Chem. Mater.* **29**, 950-957 **(2017).**
8. Kim, J., Shih, P. C., Tsao, K. C., Pan, Y. T., Yin, X., Sun, C. J. & Yang, H. High-performance pyrochlore-type yttrium ruthenate electrocatalyst for oxygen evolution reaction in acidic media. *J. Am. Chem. Soc.* **139**, 12076-12083 (2017).
9. Li, G. Q., Li, S. T., Ge, J. J., Liu, C. P. & Xing, W. Discontinuously covered IrO_2–RuO_2@Ru electrocatalysts for the oxygen evolution reaction: How high activity and long-term durability can be simultaneously realized in the synergistic and hybrid nano-structure. *J. Mater. Chem. A* **5**, 17221-17229 (2017).
10. Hu, F., Zhu, S., Chen, S., Li, Y., Ma, L., Wu, T., Zhang, Y., Wang, C., Liu, C., Yang, X.,

Song, L., Yang, X. & Xiong, Y. Amorphous metallic NiFeP: A conductive bulk material achieving high activity for oxygen evolution reaction in both alkaline and acidic media. *Adv. Mater.* **29**. 1606570 (2017).

11. Pi, Y., Shao, Q., Wang, P., Guo, J. & Huang, X. General formation of monodisperse IrM (M = Ni, Co, Fe) bimetallic nanoclusters as bifunctional electrocatalysts for acidic overall water splitting. *Adv. Func. Mater.* **27**, 1700886 (2017).
12. Kwon, T., Hwang, H., Sa, Y. J., Park, J., Baik, H., Joo, S. H. & Lee, K. Cobalt assisted synthesis of IrCu hollow octahedral nanocages as highly active electrocatalysts toward oxygen evolution reaction. *Adv. Func. Mater.* **27**, 1604688 (2017).
13. Lettenmeier, P., Wang, L., Golla-Schindler, U., Gazdzicki, P., Cañas, N. A., Handl, M., Hiesgen, R., Hosseiny, S. S., Gago, A. S. & Friedrich, K. A. Nanosized IrO_x–Ir catalyst with relevant activity for anodes of proton exchange membrane electrolysis produced by a cost-effective procedure. *Angew. Chem. Int. Ed.* **55**, 752-756 (2016).
14. Seitz, L. C., Dickens, C. F., Nishio, K., Hikita, Y., Montoya, J., Doyle, A., Kirk, C., Vojvodic, A., Hwang, H. Y., Norskov, J. K. & Jaramillo, T. F. A highly active and stable IrOx/$SrIrO_3$ catalyst for the oxygen evolution reaction. *Science* **353**, 1011-1104 (2016).
15. Diaz-Morales, O., Raaijman, S., Kortlever, R., Kooyman, P. J., Wezendonk, T., Gascon, J., Fu, W. T. & Koper, M. T. Iridium-based double perovskites for efficient water oxidation in acid media. *Nat. Commun.* **7**, 12363 (2016).
16. Reier, T., Pawolek, Z., Cherevko, S., Bruns, M., Jones, T., Teschner, D., Selve, S., Bergmann, A., Nong, H. N., Schlogl, R., Mayrhofer, K. J. & Strasser, P. Molecular insight in structure and activity of highly efficient, low-Ir Ir−Ni oxide catalysts for electrochemical water splitting (OER). *J. Am. Chem. Soc.* **137**, 13031-13040 (2015).
17. Nong, H. N., Oh, H. S., Reier, T., Willinger, E., Willinger, M. G., Petkov, V., Teschner, D. & Strasser, P. Oxide-supported $IrNiO_x$ core-shell particles as efficient, cost-effective, and stable catalysts for electrochemical water splitting. *Angew. Chem. Int. Ed.* **54**, 2975-2979 (2015);
18. Kotz, R., Lewerenz, H. J. & Stucki, S. XPS studies of oxygen evolution on Ru and RuO_2 anodes. *J. Electrochem. Soc.* **130**, 825-829 (1983).
